# Complex Reassortment Dynamics of H9N2 Avian Influenza Viruses in Xinjiang, China: Implications for Zoonotic Spillover

**DOI:** 10.1111/irv.70170

**Published:** 2025-10-20

**Authors:** Nana Chang, Haiyang Wang, Kamila Aisaiti, Jingxia Guo, Tong Wu, Cheng Zhang, Han Du, Fei Du, Yuhai Bi, Zhenghai Ma

**Affiliations:** ^1^ Xinjiang Key Laboratory of Biological Resources and Genetic Engineering, College of Life Science and Technology Xinjiang University Urumqi China; ^2^ Institute of Traditional Chinese Medicine Health Industry China Academy of Chinese Medical Sciences Nanchang China; ^3^ Disease Prevention and Control Center of Xinjiang Production and Construction Corps Urumqi Xinjiang China; ^4^ CAS Key Laboratory of Pathogenic Microbiology and Immunology, Institute of Microbiology Chinese Academy of Sciences Beijing China; ^5^ Xinjiang Second Medical College Karamay China

**Keywords:** Avian influenza virus, H9N2, molecular evolution, reassortment, zoonotic potential

## Abstract

**Background:**

H9N2 avian influenza viruses (AIVs) donate their genes to other subtype AIVs, posing significant threats to poultry industries and public health due to their endemicity and zoonotic potential. This study investigates the molecular evolution, reassortment, and mutations of the H9N2 isolates from the live poultry markets (LPMs) in Xinjiang, China.

**Methods:**

AIVs were isolated from oropharyngeal and cloacal swabs, as well as environmental samples collected during the winter of 2017–2018. Full‐genome sequencing and phylogenetic and molecular analysis were conducted to elucidate viral origins, reassortment patterns, and molecular characteristics.

**Results:**

Thirty H9N2 isolates were obtained, all belonging to the G57 genotype. Phylogenetic analysis revealed three distinct Eurasian lineages: BJ/94‐like (HA, NA), G1‐like (PB2, MP), and F98‐like. Notably, viral genes diverged into two major branches (A/B), with the A branches of HA, PB2, PA, and NS further subdivided into A1/A2 sublineages. In addition to the A and B branches, the viral genes of several isolates formed independent phylogenetic branches. Some of the viral genes clustered together with H9N2 viruses from the poultry/environmental strains in China and Japan, and some viral genes (e.g., *PB2*, *PB1*, *MP*, and *NS*) showed close phylogenetic relationships with human‐infecting H9N2/H7N9 viruses. The multiple mutations detected in the isolates were associated with viral virulence, mammalian adaptation, and transmission.

**Conclusion:**

Xinjiang H9N2 viruses display complex reassortment dynamics involving multiple geographic lineages. Their genetic connection to human‐infecting strains underscores the risk of zoonotic spillover. Enhanced surveillance in LPMs is crucial for pandemic preparedness.

## 1 Introduction

H9N2 subtype avian influenza viruses (AIVs) are commonly found among domestic poultry and wild bird populations in Asia, the Middle East, and North and West Africa since their emergence in southern China (1994) [1]. Data from the Center for Influenza Research and Early‐Warning of the Chinese Academy of Sciences indicate that H9N2 has emerged as the leading AIV subtype in domestic poultry in China from 2016 to 2023, surpassing H5N6 and H7N9 [2, 3]. Notably, H9N2 AIVs can infect domestic poultry and wild birds, and they can also infect various mammals due to their enhanced binding affinity for human‐type sialic acid receptors (α2,6‐SA), such as pigs, dogs, cats, foxes, pikas, and humans [4]. According to the World Health Organization, as of July 2022, more than 100 cases of human infection with H9N2 had been reported, with more than 50 cases occurring after 2020 [5], posing significant threats to public health. Although H9N2 viruses show low pathogenicity in birds, they can still serve as a genetic source for some reassortants that can infect humans, including H5N6, H7N9, H10N8, H10N3 [6], and the recently emerged H3N8 viruses [7]. The continuous emergence of novel viruses highlights the fact that AIVs with H9N2‐derived internal genes have an increased ability to infect humans.

Phylogenetically, H9N2 viruses are divided into North American and Eurasian lineages based on the phylogeny of the HA gene sequences. The Eurasian lineage can be further divided into different sublineages: A/chicken/Beijing/1/1994 (BJ/94‐like) or A/duck/Hong Kong/Y280/1997 (Y280‐like), A/quail/Hong Kong/G1/1997 (G1‐like), A/duck/Hong Kong/Y439/1997 (Y439‐like), A/chicken/Shanghai/F/1998 (F/98‐like), and others [8]. The BJ/94‐like subgroup predominates in northern China, whereas the G1‐like subgroup is more common in southern China [9, 10]. Before 2010, the prevalence of H9N2 viruses in China was characterized by high genetic diversity, with multiple genotypes co‐circulating [11, 12]. However, the G57 genotype of the BJ/94‐like lineage of H9N2 AIVs emerged in 2007 and became the predominant genotype in birds across China after 2010, replacing other genotypes [13]. Notably, since 2013, the G57 H9N2 viruses have possessed the backbone of F/98‐like viruses and incorporated the polymerase basic protein 2 (PB2) and matrix (M) genes from G1‐like viruses [14]. Importantly, the G57 H9N2 virus can not only infect humans directly but also provide partial or complete sets of internal genes for emerging human‐infecting AIVs, such as H3N8, H7N9, H10N8, and H10N3 reassortants [6, 7]. Given their endemicity and zoonotic potential, these viruses pose significant threats to both the poultry industry and public health. Therefore, continuous monitoring of the evolution of H9N2 viruses is essential to control their spread. The study characterizes 30 H9N2 isolates from Urumqi LPMs, revealing their evolutionary origins, reassortment events, and mammalian‐adaptation mutations. The findings underscore the role of LPMs as hubs for viral evolution and interspecies transmission.

## 2 Materials and Methods

### 2.1 Sample Collection

From January 2017 to December 2018, 973 oropharyngeal and cloacal swabs (from chickens, ducks, and geese) and 68 environmental samples (including feces, cages, and wastewater) were collected monthly from Urumqi Live Poultry Markets (LPMs). The samples were stored in viral transport medium (with a pH of 7.0–7.5) at −80°C posttransport.

### 2.2 Virus Isolation and Identification

The collected swabs underwent three freeze–thaw cycles, followed by centrifugation at 7000 rpm for 10 min at 4°C to obtain the supernatant. The supernatant was then filtered through a 0.22‐μm filter, and 100 μL of the filtered supernatant was inoculated into 10‐day‐old SPF embryonated eggs. Hemagglutination‐positive samples underwent RT‐PCR targeting the PB1 gene [15]. Viral RNA extraction (Simply P Virus RNA Extraction Kit, Bioer) and RT‐PCR (RT‐PCR Quick Master Mix, TOYOBO) were performed according to the manufacturers' instructions. All experiments adhered to BSL‐2/BSL‐3 protocols.

### 2.3 Whole‐Genome Sequencing

Next‐generation sequencing (NGS) was utilized to ascertain the whole‐genome sequences of the AIV isolates. The viral RNA samples were quantified employing the 2100 Bioanalyzer System (Agilent Technologies). RT‐PCR and complementary DNA (cDNA) synthesis were conducted using the PrimeScript One‐Step RT‐PCR Kit Ver.2 (RR055A, Takara) and influenza A–specific primers MBTuni‐12 and MBTuni‐13 [16]. The full‐length PCR amplification of eight viral gene segments was combined to construct a DNA library for high‐throughput sequencing for each sample. The combined PCR products were fragmented by sonication using a Covaris LE220 Focused‐ultrasonicator (Covaris) [17]. DNA libraries with an insert size of 200 bp were prepared through end‐repairing, dA‐tailing, adaptor ligation, and PCR amplification, all in accordance with the standard manufacturer's instructions (Illumina USA). The libraries were sequenced on an Illumina HiSeq4000 platform (Illumina) [18].

### 2.4 Sequencing Data Assembly

The raw NGS reads were cleaned by filtering out low‐quality reads (eight bases with quality < 66), adaptor‐contaminated reads (with > 15 bp matched to the adaptor sequence), poly‐Ns (with 8Ns), duplication, and host‐contaminated reads [19]. The clean reads were mapped to the INFLUENZA database [20] to obtain the best‐matching reference sequences. Burrows‐Wheeler Aligner (BWA version 0.5.9) and SAMtools (version 0.1.19) [19] were used to perform assembly based on the reference sequences.

### 2.5 Phylogenetic Analysis and Molecular Characterization

Phylogenetic trees were constructed for each gene segment of all H9N2 isolates to investigate their evolutionary relationships. The AIV reference sequences were obtained from GenBank (http://www.ncbi.nlm.nih.gov/genbank) and GISAID (http://www.gisaid.org) using the online Basic Local Alignment Search Tool (BLAST). The selected reference sequences encompassed representative strains from the major circulating lineages in China, including A/Chicken/Beijing/1/94, A/Duck/Hong Kong/Y280/97, A/Chicken/Shanghai/F/98, A/Quail/Hong Kong/G1/97, and A/Turkey/Wisconsin/1/1966. Furthermore, other relevant strains with sequence identities of ≥ 99% were included to ensure complete coverage and accurate phylogenetic analysis, with only one strain retained from the same cluster if it was isolated in the same year, from the same host, and location. The isolates and reference strains for the eight gene segments are detailed in Tables S1 and S2, respectively. Phylogenetic trees were constructed using the neighbor‐joining (NJ) method with bootstrap analysis involving 1000 replications in the MEGA X software. The evolutionary distances were computed using the Tamura‐Nei model as the number of base‐pair substitutions per nucleotide site.

## 3 Results

### 3.1 Virus Isolation

Between January 2017 and December 2018, 30 isolates of H9N2 AIV were obtained from the LPM in Urumqi, Xinjiang. These isolates included 26 from chickens, 3 from ducks, and 1 from the environment. They have been abbreviated as XJ/01‐021/17 and XJ/022‐030/18, and their nucleotide sequences have been submitted to the Global Initiative on Sharing All Influenza Data (GISAID). (See Table S1 for details).

### 3.2 Genetic Identity Analysis

BLAST analysis revealed that the HA genes of 30 isolates were closely related, exhibiting nucleotide identities ranging from 89.8% to 100%. Similarly, the NA genes shared nucleotide identities from 85.7% to 100%. The six internal viral genes—PB2, PB1, PA, NP, MP, and NS—showed nucleotide identities ranging from 95.4% to 100%, 94.1% to 100%, 94.9% to 100%, 95.2% to 100%, 95.9% to 100%, and 95.2% to 100%, respectively. When aligned with reference isolates from the NCBI and GISAID databases, the HA genes demonstrated the highest sequence identity (95.97%–99.41%) with A/Duck/Jiangxi/X2368/2017(H9N2), A/chicken/Xuzhou/XZ270/2016(H9N2), A/chicken/Ningxia/NX2203/2017(H9N2), and A/chicken/China/355/2017(H9N2). The NA genes had the highest sequence identity (98.1%–98.9%) with A/Duck/China/F3143/2015(H9N2), A/Hubei/295/2019(H9N2), A/chicken/JinShui/JS1002/2018(H9N2), and A/chicken/China/93/2017(H9N2). The six internal genes exhibited the highest sequence identities with H7N9 and H9N2 isolates from chicken flocks in Northern China (Hebei and Tianjin), Central China (Henan, Hubei, and Hunan), Eastern China (Anhui, Jiangsu, and Zhejiang), and Southern China (Guangdong) from 2014 to 2017.

### 3.3 Phylogenetic Analysis

**FIGURE 1:**
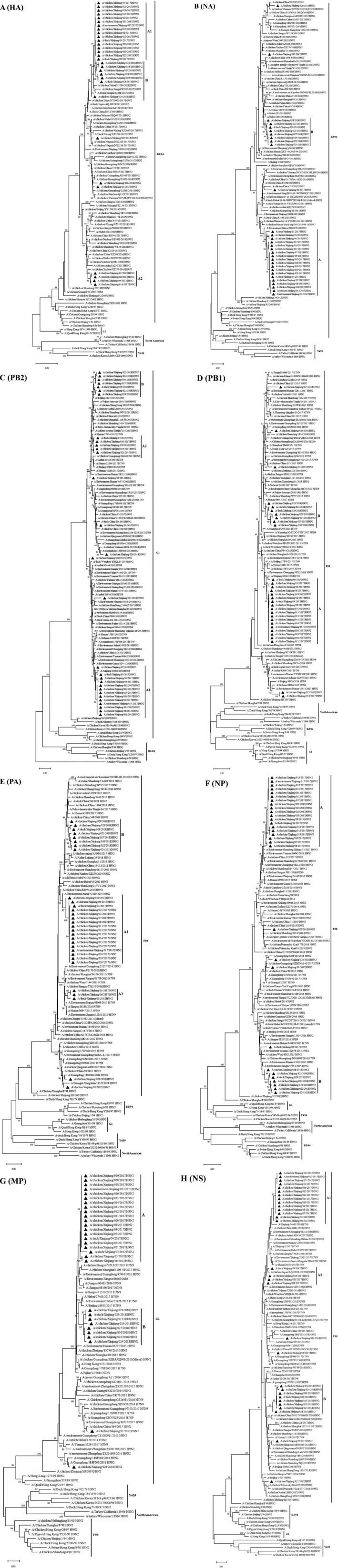
Phylogenetic analysis of *HA* (A), *NA* (B), *PB2* (C), *PB1* (D), *PA* (E), *NP* (F), *MP* (G), and *NS* (H) gene segments of the H9N2 isolated from Xinjiang. Multiple alignments were constructed using Clustal W. Phylogenetic trees were generated using the neighbor‐joining method (NJ) with 1000 bootstrap replicates in the MEGA X program. H9N2 isolates are depicted by triangles.

Phylogenetic trees for the viral eight genes were generated as depicted in Figure 1A–H. Phylogenetic analysis indicated that all isolates were classified as genotype G57 viruses, with their gene segments belonging to three distinct Eurasian lineages: The HA and NA genes were part of the BJ/94‐like lineage, the PB2 and MP genes belonged to the G1‐like lineage, and the remaining four genes were associated with the F98‐like lineage (Figure 1). Despite all isolates being of the genotype G57, the viral genes still displayed diversity.

Based on the year of isolation and clustering patterns, the H9N2 isolates were primarily categorized into two distinct branches labeled A (XJ/02‐021/17) and B (XJ/022‐025/18 and XJ/027‐030/18). Furthermore, the A branches of HA, PB2, PA, and NS were subdivided into two smaller subbranches (A1 and A2) (Figure 1). The A and B branches of HA, PA, and NP; the A branch of NA; and the A2 subbranch and B branch of NS all clustered with H9N2 viruses predominantly isolated from poultry and the environment in China (including Jiangsu, Anhui, Shandong, Jiangxi, Hubei, Hunan, and Gansu) and Japan; the A branch of MP clustered with H7N9 and H9N2 viruses from poultry in Jiangsu and Shanghai; the A and B branches of PB2 and PB1, the B branch of MP, and the A1 subbranch of NS all clustered with human‐infecting H7N9 viruses circulating in Xinjiang, Henan, Hebei, Hunan, and Beijing; and the B branch of NA clustered with human‐infecting H9N2 viruses from Hubei and Hunan. Apart from the A and B branches, the viral genes of several isolates (including XJ/01/17, XJ/03/17, XJ/09/17, XJ/012/17, XJ/014/17, XJ/025/18, and XJ/026/18) formed independent phylogenetic branches. Some of these (such as the PA of XJ/01/17 and all 6 internal genes of XJ/026/18) clustered with human‐infecting H9N2 viruses from Guangdong, Guangxi, and Hunan; others (such as the NS of XJ/01/17 and the PB1 of XJ/012/17 and XJ/014/17) clustered with human‐infecting H7N9 viruses from Guangdong, Beijing, and Sichuan; and the remaining genes clustered with H9N2 and H7N9 viruses isolated from poultry in China.

### 3.4 Molecular Characteristics

The HA cleavage sites of the isolates contained only one basic amino acid (PSRSSR/GLF), characteristic of LPAIV. Mutations at the receptor‐binding sites of the HA proteins, such as Q226L, I155T, H183N, and A190T (H3 numbering), were found in the isolates (Table S3), suggesting a preference for human α2,6‐linked sialic acid (α2‐6‐SA) receptor binding. In the PB2 proteins, 12 isolates contained the E627V mutation, and the A588V mutation was present in all isolates except XJ/01/2017. Additionally, mutations that could enhance viral replication, virulence, or transmission in mammalian hosts were identified in the isolates, including K356R, N383D, and A515T in PB1, N30D and T215A in PA, and P42S and V149A in M1 (Table S3).

## 4 Discussion

LPMs play a pivotal role in the emergence of novel AIVs by facilitating interspecies transmission and genetic reassortment [21, 22]. H9N2 AIVs, as endemic pathogens in global poultry production systems [23, 24, 25], not only impair egg production but also act as prolific donors of internal genes to other AIV subtypes. Notably, reassortment events involving H9N2 have generated highly pathogenic AIVs (HPAIVs) such as H5N6, H5N8, and H7N9, posing dual threats to food security and public health [18, 26, 27, 28]. In Xinjiang, previous surveillance identified H5N6, H5N8, and H5N2 HPAIVs in the same LPM, all of which resulted from complex reassortment among H5Nx, H7N9, and H9N2 viruses, with internal genes traced back to local H9N2 strains [29, 30, 31, 32]. This work confirms the dominance of the G57 genotype among Xinjiang H9N2 viruses, consistent with its prevalence in Chinese poultry since 2010 [33]. Phylogenetic analysis has revealed a tri‐reassortant structure that includes lineages similar to BJ/94 (HA, NA), G1 (PB2, MP), and F98 (PB1, NP, PA, NS). H9N2 viruses indicate the genetic diversity and complexity of the H9N2 subtype AIVs in Xinjiang. Similarly, multiple reassortment events have been detected in several provinces, including Guangdong, Guangxi, Yunnan, and Jiangxi. The genetic mosaicism within H9N2 viruses is a manifestation of their dynamic evolution, driven by selective pressures from immunological, ecological, and agricultural factors. Specifically, our research has identified an interspecies transmission pattern of G57 H9N2 isolates in LPMs, where chickens serve as the primary hosts, followed by domestic ducks. This bidirectional transmission pattern between chickens and ducks has been confirmed in previous studies and could further facilitate the emergence of novel viruses within the H9N2 lineage. This is a cause for concern, as a similar bidirectional transmission event has been identified in the evolution and global spread of H5N1 viruses within the Goose/Guangdong/1996 (GS/96) lineage. This suggests that the interspecies transmission dynamics may pose a significant risk for the generation of new viral strains with potentially broader host ranges and greater public health implications.

Phylogenetic analysis also revealed that the internal genes of Xinjiang H9N2 isolates (PB1, PB2, NS, and MP) formed distinct clusters with human‐infecting H7N9 viruses from Xinjiang (e.g., A/Xinjiang/04062/2018) and multiple other provinces (Henan, Hebei, Beijing, Hunan, Guangdong, and Sichuan). Epidemiological data indicated 14 human H7N9 cases in Xinjiang by January 2018, with most cases being linked to LPM exposure. Notably, the PB1‐A branch, PB2‐A1 sublineage, and NS‐A1 sublineage of H9N2 isolates exhibited near‐identical nucleotide identities (ranging from 98.6% to 100%) and clustered together with the corresponding genes of the 2018 Xinjiang H7N9 strain (Figure 1), aligning with previous reports on the viral genes (including NA, PB1, PB2, PA, and NP) of the reassortant H5N2 HPAIVs in Urumuqi, which originated from the H9N2 isolates in the same LPM [29]. These findings strongly suggest that the H9N2 AIVs were circulating at the avian‐human interface and donated their genes to generate novel reassortment AIVs persistently in Urumuqi and bidirectional gene flow between H9N2 and H7N9 viruses within the LPM environment. These findings corroborate that H9N2 AIVs are circulating at the avian‐human interface [34]. The first documented human infections with H9N2 AIVs occurred in Hong Kong in 1999 [35]. As of July 2022, over 100 cases of human infection with H9N2 AIVs have been reported [1]. The G57 H9N2 virus not only infects humans directly but also contributes a complete set or partial internal genes to new reassortants. Multiple human‐infecting AIVs, including H7N9, H5N6, H10N8, H10N3 [6], and the H3N8 subtype, which was first identified in Henan Province in April 2022 [7], have been recognized to carry internal genes from the G57 H9N2 virus, posing significant public health concerns. Clearly, the endemic and widespread prevalence of the G57 H9N2 virus in poultry provides an optimal environment for viruses of different subtypes to exchange their gene segments with H9N2 viruses. The study indicated NB‐A and PA‐A branches of XJ/01/17, and all six internal genes of XJ/026/18 clustered with human‐infecting H9N2 AIVs from Hubei, Hunan, Guangdong, and Guangxi. Human H9N2 infections are generally mild and easily overlooked in these settings, which facilitates further adaptation in humans. This occurs through reassortment with other AIVs and the generation of novel AIV subtypes that could possess high reproductive capacity and even efficient transmissibility [14, 26, 36]. The first human infection with a novel avian‐origin H7N9 AIV was reported in eastern China in March 2013 [14]. The H7N9 AIVs originated from a reassortment between H7N9 viruses from wild birds (supplying the surface genes) and poultry H9N2 viruses (providing the internal genes). These viruses have caused severe respiratory symptoms and mortality. Previous studies indicate that the H7N9 viruses responsible for human infections since 2013 have been directly linked to the LPMs. The viral internal genes were derived from H9N2 viruses [37, 38], and certain H9N2‐derived genes have increased the polymerase activity of the H7N9 subtype virus in human cells [39].

We found that the viral genes of the H9N2 isolates were from multiple geographic origins, encompassing regions such as Xinjiang, Eastern China, Southern China, Northern China, Central China, Japan, and more. Previous studies indicated that the H5N8, H5N6, and H5N2 AIVs isolated in the same LPM in Urumuqi also involved multiple geographic introductions and reassortment events, and migratory birds carrying multi‐subtype AIVs could spread these viruses into Xinjiang and transmit them to aquatic poultry, subsequently spreading into local LPMs [29, 30, 31, 32]. The results indicated that some genes of the H9N2 isolates may originate from AIVs carried by migratory birds. Additionally, the trans‐regional poultry trade facilitates the spread of the H9N2 virus. Notably, Eastern and Southern China are home to numerous poultry breeding areas and bustling poultry markets. Furthermore, this study revealed that the partial genes of the H9N2 isolates, and indeed, the majority of genes in some H9N2 isolates (such as XJ/026/18) cluster with those of local AIV isolates.

In our study, the multiple mutations identified in the H9N2 isolates were associated with viral virulence, adaptation, and transmission (Table S3), and these mutations had been found in the human H9N2 isolates [35, 40, 41, 42, 43]. In the viral HA protein, the cleavage sites contained one basic amino acid (PSRSSR/GLF), which represents LPAIV [44]. The receptor‐binding sites in the HA protein all contained 226L (H3 numbering), suggesting that the isolates preferentially bind to the human‐like α‐2,6‐SA receptor [45]. Previous surveillance data have indicated that the Q226L mutation in the HA protein is present in over 75% of the H9N2 viruses currently circulating in mainland China. This site substitution can increase viral replication and facilitate direct contact transmission among ferrets under experimental conditions [46, 47]. Moreover, the I155T, H183N, and T190A mutations could also enhance human‐type sialic acid receptor affinity of the H9N2 isolates from Xinjiang and promote viral infection in human cells and transmission in mammals [48, 49]. In the NA stalk region, the isolates all had three amino acid deletions (62–64) associated with the adaptation of wild‐bird‐origin AIVs to poultry [50]. Therefore, it is essential to closely monitor the H9N2 virus in wild birds to minimize the risk of its further widespread dissemination. The ribonucleoprotein (RNP) complex, which consists of the PB2, PB1, PA, and NP proteins, is vital for viral replication, virulence, and the cross‐species transmission of viruses from birds to mammals. The A588V mutation was identified in all isolates except for XJ/01/17, and the E627V mutation was found in 12 isolates of the PB2 protein, which enhances viral replication in mammalian cells and increases pathogenicity in mouse models [3]. A recent study has indicated that the number of strains carrying the A588V mutation has increased since 2016, and the proportion of E627V has also risen from 2021 to 2023 [3]. Additionally, the E627V mutation has been detected in human‐infecting H9N2 viruses, specifically in A/Beijing/1/2016 and A/Beijing/1/2017 [51, 52]. L89V, G309D, R477G, and I495V mutations in the PB2 [53]; L13P [54] and I368V [55] mutations in the PB1; K356R [56], N383D, and A515T mutations in the PA [57]; N30D and T215A mutations in the M1 [58]; P42S [59] and V149A [60] mutations in the NS1 were detected in all isolates. These mutations are known to be associated with mammalian host specificity and increased virulence in ferrets and mice.

## 5 Conclusions

This study elucidates the tri‐reassortant nature of Xinjiang H9N2 viruses and their genetic linkage to human‐infecting strains. Continuous surveillance and molecular characterization are imperative to preempt zoonotic spillover events.

## Author Contributions

**Nana Chang:** methodology, software, data curation, investigation, visualization, formal analysis, writing – original draft, writing – review and editing. **Haiyang Wang:** methodology, software, investigation, writing – original draft, visualization, data curation, formal analysis. **Kamila Aisaiti:** methodology, software, data curation, investigation, formal analysis, visualization, writing – original draft. **Jingxia Guo:** methodology, investigation. **Tong Wu:** methodology, investigation. **Cheng Zhang:** methodology, investigation, validation. **Han Du:** methodology, software, investigation, formal analysis. **Fei Du:** methodology, software, investigation, formal analysis, methodology, investigation. **Yuhai Bi:** conceptualization, writing – review and editing. **Zhenghai Ma:** conceptualization, methodology, investigation, formal analysis, validation, supervision, visualization, funding acquisition, project administration, resources, writing – review and editing.

## Conflicts of Interest

The authors declare no conflicts of interest.

## Peer Review

The peer review history for this article is available at https://www.webofscience.com/api/gateway/wos/peer‐review/10.1111/irv.70170.

## Supporting information


**Table S1:** Species origin, geographic distribution, isolation time, and GenBank accession numbers of H9N2 isolates.

**Table S2:** Detailed information of the eight genes of the reference sequence used for phylogenetic analysis.

**Table S3:** Amino acid mutations identified in the H9N2 isolates contribute to increased viral binding to human‐type receptors, replication, virulence, or transmission in mammals.

## Data Availability

The viral sequences from this study are available on GISAID and NCBI, which are provided in the Supporting Information.
