# Supplementary material for: Complex Reassortment Dynamics of H9N2 Avian Influenza Viruses in Xinjiang, China: Implications for Zoonotic Spillover
Source: Influenza Other Respir Viruses. 2025 Oct 20;19(10):e70170. doi: 10.1111/irv.70170 (PMC12537064; doi:10.1111/irv.70170)
Supplement: Supplementary file 1 — Table S1: Species origin, geographic distribution, isolation time, and GenBank accession numbers of H9N2 isolates. [file IRV-19-e70170-s003.docx]

| **Supplementary Table 1. The reference sequences of H9N2 AIVs** | | | |
| --- | --- | --- | --- |
| HA | Strain name | Subtype | Isolate ID |
| GISAID | A/chicken/Hubei/H1080/2018 | H9N2 | EPI_ISL_374132 |
|  | A/chicken/Jiangxi/X2355/2017 | H9N2 | EPI_ISL_375935 |
|  | A/Duck/Jiangxi/X2368/2017 | H9N2 | EPI_ISL_375936 |
|  | A/chicken/Daye/DY0602/2017 | H9N2 | EPI_ISL_281313 |
|  | A/duck/Japan/AQ-HE28/2015 | H9N2 | EPI_ISL_253948 |
|  | A/chicken/Ganzhou/GZ126/2016 | H9N2 | EPI_ISL_252840 |
|  | A/Duck/China/E552/2014 | H9N2 | EPI_ISL_501407 |
|  | A/chicken/Sichuan/SZQ60/2015 | H9N2 | EPI_ISL_253021 |
|  | A/chicken/Hubei/H1038/2016 | H9N2 | EPI_ISL_371164 |
|  | A/chicken/Guangdong/G1301/2016 | H9N2 | EPI_ISL_501362 |
|  | A/chicken/Xizang/XZ2065/2017 | H9N2 | EPI_ISL_375963 |
|  | A/Duck/Xizang/XZ2239/2017 | H9N2 | EPI_ISL_376007 |
|  | A/chicken/Ningxia/NX2203/2017 | H9N2 | EPI_ISL_375668 |
|  | A/chicken/Ningxia/NX2216/2017 | H9N2 | EPI_ISL_375672 |
|  | A/Environment/Xinjiang/39018/2015 | H9N2 | EPI_ISL_329998 |
|  | A/chicken/Guizhou/Q1303/2018 | H9N2 | EPI_ISL_374391 |
|  | A/Duck/Guangdong/G2041/2017 | H9N2 | EPI_ISL_375384 |
|  | A/chicken/Guangdong/G2236/2017 | H9N2 | EPI_ISL_375431 |
|  | A/chicken/Guangdong/G2479/2016 | H9N2 | EPI_ISL_371553 |
|  | A/chicken/China/H1027/2017 | H9N2 | EPI_ISL_501369 |
|  | A/chicken/Guangdong/G1040/2018 | H9N2 | EPI_ISL_374113 |
|  | A/chicken/Guangdong/G1051/2018 | H9N2 | EPI_ISL_314960 |
|  | A/chicken/Guangdong/G2068/2017 | H9N2 | EPI_ISL_375388 |
|  | A/chicken/Guizhou/Q1323/2018 | H9N2 | EPI_ISL_374409 |
|  | A/chicken/Vietnam/NCVD-H5C18LS10/2018 | H9N2 | EPI_ISL_408140 |
|  | A/chicken/Jiangsu/J2154/2018 | H9N2 | EPI_ISL_374800 |
|  | A/chicken/Shanghai/S2134/2018 | H9N2 | EPI_ISL_375071 |
|  | A/chicken/Beijing/XY1206/2014 | H9N2 | EPI_ISL_378670 |
|  | A/chicken/Shandong/1875/2019 | H9N2 | EPI_ISL_502324 |
|  | A/chicken/ShanXi/1778/2019 | H9N2 | EPI_ISL_502328 |
|  | A/chicken/Xizang/XZ2102/2018 | H9N2 | EPI_ISL_375249 |
|  | A/chicken/Jiangxi/X1081/2018 | H9N2 | EPI_ISL_374527 |
|  | A/Hubei/2081/2019 | H9N2 | EPI_ISL_499110 |
|  | A/chicken/China/H1301/2017 | H9N2 | EPI_ISL_501362 |
|  | A/chicken/Guizhou/GZ1063/2016 | H9N2 | EPI_ISL_371330 |
|  | A/chicken/Shandong/3430/2017 | H9N2 | EPI_ISL_502188 |
|  | A/chicken/Shandong/WF39/2016 | H9N2 | EPI_ISL_372501 |
|  | A/chicken/China/F2219/2015 | H9N2 | EPI_ISL_501340 |
|  | A/chicken/China/XZ299/2016 | H9N2 | EPI_ISL_501340 |
|  | A/chicken/Huibei/H2197/2016 | H9N2 | EPI_ISL_371394 |
|  | A/chicken/Guizhou/Q2381/2016 | H9N2 | EPI_ISL_371620 |
|  | A/chicken/Anhui/A230/2017 | H9N2 | EPI_ISL_374020 |
|  | A/chicken/Xuzhou/XZ270/2016 | H9N2 | EPI_ISL_284665 |
|  | A/chicken/Shandong/JN/1999 | H9N2 | EPI_ISL_77230 |
|  | A/chicken/Guangxi/55/2005 | H9N2 | EPI_ISL_13013 |
|  | A/chicken/Zhejiang/HJ/2007 | H9N2 | EPI_ISL_63471 |
|  | A/chicken/Shantou/3173/2001 | H9N2 | EPI_ISL_7488 |
|  | A/chicken/Guangdong/FZH/2011 | H9N2 | EPI_ISL_89721 |
|  | A/Duck/Hong Kong/Y280/97 | H9N2 | EPI_ISL_1266 |
|  | A/Chicken/Hong Kong/G9/97 | H9N2 | EPI_ISL_146698 |
|  | A/Chicken/Guangdong/SS/94 | H9N2 | EPI_ISL_68726 |
|  | A/Chicken/Shanghai/F/98 | H9N2 | EPI_ISL_68579 |
|  | A/Chicken/Beijing/1/94 | H9N2 | EPI_ISL_146731 |
|  | A/Chicken/Shandong/6/96 | H9N2 | EPI_ISL_2240 |
|  | A/Hong Kong/1074/1999 | H9N2 | EPI_ISL_302716 |
|  | A/Quail/Hong Kong/G1/97 | H9N2 | EPI_ISL_260531 |
|  | A/chicken/Heilongjiang/35/00 | H9N2 | EPI_ISL_8504 |
|  | A/turkey/Wisconsin/1/1966 | H9N2 | EPI_ISL_132848 |
|  | A/Turkey/California/189/66 | H9N2 | EPI_ISL_1280 |
|  | A/duck/Hong Kong/784/1979 | H9N2 | EPI_ISL_1273 |
|  | A/Duck/Hong Kong/Y439/97 | H9N2 | EPI_ISL_146723 |
|  | A/chicken/Korea/MS96-CE6/1996 | H9N2 | EPI_ISL_68689 |
| NCBI | A/chicken/China/A47/2018 | H9N2 | MK806537 |
|  | A/chicken/China/355/2017 | H9N2 | MN385379 |
|  | A/chicken/China/59/2017 | H9N2 | MG847101 |
|  | A/chicken/Xinjiang/01/2017 | H9N2 | MW097419 |
|  | A/chicken/Xinjiang/02/2017 | H9N2 | MW097420 |
|  | A/duck/Xinjiang/03/2017 | H9N2 | MW097421 |
|  | A/duck/Xinjiang/04/2017 | H9N2 | MW097422 |
|  | A/chicken/Xinjiang/05/2017 | H9N2 | MW106992 |
|  | A/chicken/Xinjiang/06/2017 | H9N2 | MW107000 |
|  | A/chicken/Xinjiang/07/2017 | H9N2 | MW097424 |
|  | A/chicken/Xinjiang/08/2017 | H9N2 | MW097425 |
|  | A/chicken/Xinjiang/09/2017 | H9N2 | MW097426 |
|  | A/chicken/Xinjiang/010/2017 | H9N2 | MW097427 |
|  | A/chicken/Xinjiang/011/2017 | H9N2 | MW106944 |
|  | A/chicken/Xinjiang/012/2017 | H9N2 | MW097428 |
|  | A/chicken/Xinjiang/013/2017 | H9N2 | MW097429 |
|  | A/chicken/Xinjiang/014/2017 | H9N2 | MW097430 |
|  | A/chicken/Xinjiang/015/2017 | H9N2 | MW106960 |
|  | A/chicken/Xinjiang/016/2017 | H9N2 | MW106952 |
|  | A/environment/Xinjiang/017/2017 | H9N2 | MW097431 |
|  | A/chicken/Xinjiang/018/2017 | H9N2 | MW097432 |
|  | A/chicken/Xinjiang/019/2017 | H9N2 | MW097433 |
|  | A/chicken/Xinjiang/020/2017 | H9N2 | MW097434 |
|  | A/chicken/Xinjiang/021/2017 | H9N2 | MW097435 |
|  | A/chicken/Xinjiang/022/2018 | H9N2 | MW097588 |
|  | A/chicken/Xinjiang/023/2018 | H9N2 | MW097589 |
|  | A/chicken/Xinjiang/024/2018 | H9N2 | MW106976 |
|  | A/chicken/Xinjiang/025/2018 | H9N2 | MW097590 |
|  | A/chicken/Xinjiang/026/2018 | H9N2 | MW097591 |
|  | A/chicken/Xinjiang/027/2018 | H9N2 | MW097592 |
|  | A/chicken/Xinjiang/028/2018 | H9N2 | MW097593 |
|  | A/duck/Xinjiang/029/2018 | H9N2 | MW097594 |
|  | A/chicken/Xinjiang/030/2018 | H9N2 | MW097595 |

| NA | Strain name | Subtype | Isolate_ID |
| --- | --- | --- | --- |
| GISAID | A/chicken/Vietnam/NCVD-H5C18LS73/2018 | H9N2 | EPI_ISL_408131 |
|  | A/chicken/China/H1027/2017 | H9N2 | EPI_ISL_501369 |
|  | A/Guangdong/18SF064/2018 | H9N2 | EPI_ISL_345234 |
|  | A/Guangdong/18SF003/2018 | H9N2 | EPI_ISL_337277 |
|  | A/Guangxi-Xiangshan/11522/2018 | H9N2 | EPI_ISL_345235 |
|  | A/chicken/China/F344/2015 | H9N2 | EPI_ISL_501324 |
|  | A/pigeon/Wuxi/5997/2015 | H9N2 | EPI_ISL_277121 |
|  | A/chicken/China/F2170/2015 | H9N2 | EPI_ISL_501339 |
|  | A/chicken/Japan/AQ-HE28-28/2016 | H9N2 | EPI_ISL_280895 |
|  | A/Hunan/34179/2018 | H9N2 | EPI_ISL_345236 |
|  | A/Hubei/295/2019 | H9N2 | EPI_ISL_499117 |
|  | A/Hubei/1693/2019 | H9N2 | EPI_ISL_499108 |
|  | A/Environment/Zhenjiang/zj26/2014 | H9N2 | EPI_ISL_170169 |
|  | A/chicken/Hunan/XKY-46/2015 | H9N2 | EPI_ISL_200629 |
|  | A/environment/Fujian/08424/2013 | H9N2 | EPI_ISL_328580 |
|  | A/Environment/Guangdong/46642/2016 | H9N2 | EPI_ISL_283792 |
|  | A/chicken/Vietnam/NCVD-H5C18LS09/2018 | H9N2 | EPI_ISL_408141 |
|  | A/chicken/China/F4198/2015 | H9N2 | EPI_ISL_501345 |
|  | A/environment/JiangXi/05-07-NCJD0082C/2015 | H9N2 | EPI_ISL_198999 |
|  | A/chicken/Jiangxi/04-01-NCDZT0055-O/2015 | H9N2 | EPI_ISL_198983 |
|  | A/duck/Hubei/03-06-WHWTZ0108-P/2015-Mixed | H9N2 | EPI_ISL_200422 |
|  | A/duck/Hunan/04-14-YYGK901-O/2015 | H9N2 | EPI_ISL_199141 |
|  | A/Environment/Xinjiang/39018/2015 | H9N2 | EPI_ISL_329998 |
|  | A/Duck/China/F3143/2015 | H9N2 | EPI_ISL_501342 |
|  | A/chicken/Hunan/04-14-YYXS832-O/2015 | H9N2 | EPI_ISL_199135 |
|  | A/chicken/Hunan/YueYang0501/2015 | H9N2 | EPI_ISL_199135 |
|  | A/Environment/Gansu/01680/2016 | H9N2 | EPI_ISL_283756 |
|  | A/Quail/Hong Kong/G1/97 | H9N2 | EPI_ISL_1268 |
|  | A/Chicken/Beijing/1/94 | H9N2 | EPI_ISL_146731 |
|  | A/Chicken/Hong Kong/G9/97 | H9N2 | EPI_ISL_1270 |
|  | A/Duck/Hong Kong/Y439/97 | H9N2 | EPI_ISL_1267 |
| NCBI | A/chicken/China/93/2017 | H9N2 | MN385411 |
|  | A/Turkey/California/189/66 | H9N2 | AF156401 |
|  | A/chicken/Xinjiang/34/2012 | H9N2 | KP185557 |
|  | A/chicken/Shandong/L1/2007 | H9N2 | EU939162 |
|  | A/chicken/Zhejiang/HJ/2007 | H9N2 | FJ581434 |
|  | A/Chicken/Guangdong/SS/94 | H9N2 | DQ874395 |
|  | A/Chicken/Shandong/6/96 | H9N2 | DQ064430 |
|  | A/Duck/Hong Kong/Y280/97 | H9N2 | AF156394 |
|  | A/Swine/Hong Kong/9/98 | H9N2 | AF222812 |
|  | A/chicken/Guangxi/55/2005 | H9N2 | EU086250 |
|  | A/Chicken/Shanghai/F/98 | H9N2 | AY253754 |
|  | A/chicken/Anhui/AH329/2016 | H9N2 | MG063458 |
|  | A/chicken/Guangdong/28/2017 | H9N2 | MT032410 |
|  | A/chicken/China/355/2017 | H9N2 | MN385381 |
|  | A/environment/Zhongshan/ZS201603/2016 | H9N2 | KX783311 |
|  | A/chicken/China/G1773NA-16GD/2016 | H9N2 | MK326604 |
|  | A/Beijing/1/2017 | H9N2 | MF440738 |
|  | A/chicken/Ganzhou/GZ86/2016 | H9N2 | KY415727 |
|  | A/chicken/Zhejiang/SIC40/2015 | H9N2 | KX598510 |
|  | A/chicken/China/728/2017 | H9N2 | MN385398 |
|  | A/duck/China/D4/2018 | H9N2 | MN384778 |
|  | A/environment-air/Kunshan/NIOSH-BL25/2018 | H9N2 | MN607158 |
|  | A/chicken/China/1104/2019 | H9N2 | MN918145 |
|  | A/chicken/Shanghai/05/2018 | H9N2 | MK053848 |
|  | A/chicken/Fujian/SD037/2017 | H9N2 | MG192155 |
|  | A/chicken/China/63/2019 | H9N2 | MN263212 |
|  | A/chicken/Xuzhou/XZ270/2016 | H9N2 | MG063459 |
|  | A/chicken/Shanghai/15/2015 | H9N2 | KU720447 |
|  | A/chicken/China/1646-6/2018 | H9N2 | MK367632 |
|  | A/environment/Hunankaifu/361/2017 | H9N2 | MG220415 |
|  | A/Accipiter-gentilis-schvedowi/Tianjin/22/2017 | H9N2 | MH114052 |
|  | A/Athene-noctua/Tianjin/Y14/2017 | H9N2 | MH114060 |
|  | A/chicken/JinShui/JS1002/2018 | H9N2 | MH375878 |
|  | A/environment-air/Kunshan/NIOSH-BL34/2018 | H9N2 | MN607176 |
|  | A/chicken/Anhui/AH326/2016 | H9N2 | MG051188 |
|  | A/chicken/Heilongjiang/35/00 | H9N2 | DQ064420 |
|  | A/Chicken/Korea/38349-p96323/96 | H9N2 | AF156400 |
|  | A/turkey/Wisconsin/1/1966 | H9N2 | AB295602 |
|  | A/chicken/Jiangsu/WJ179/2015 | H9N2 | MN135927 |
|  | A/chicken/China/G1376NA-16GD/2016 | H9N2 | MK326596 |
|  | A/Hong Kong/1073/99 | H9N2 | AJ404629 |
|  | A/chicken/Shaoguan/zd201603/2017 | H9N2 | MK250369 |
|  | A/chicken/Xinjiang/01/2017 | H9N2 | MW099781 |
|  | A/chicken/Xinjiang/02/2017 | H9N2 | MW099782 |
|  | A/duck/Xinjiang/03/2017 | H9N2 | MW099783 |
|  | A/duck/Xinjiang/04/2017 | H9N2 | MW099784 |
|  | A/chicken/Xinjiang/05/2017 | H9N2 | MW106994 |
|  | A/chicken/Xinjiang/06/2017 | H9N2 | MW107002 |
|  | A/chicken/Xinjiang/07/2017 | H9N2 | MW099786 |
|  | A/chicken/Xinjiang/08/2017 | H9N2 | MW099787 |
|  | A/chicken/Xinjiang/09/2017 | H9N2 | MW099788 |
|  | A/chicken/Xinjiang/010/2017 | H9N2 | MW099789 |
|  | A/chicken/Xinjiang/011/2017 | H9N2 | MW106946 |
|  | A/chicken/Xinjiang/012/2017 | H9N2 | MW099790 |
|  | A/chicken/Xinjiang/013/2017 | H9N2 | MW099791 |
|  | A/chicken/Xinjiang/014/2017 | H9N2 | MW099792 |
|  | A/chicken/Xinjiang/015/2017 | H9N2 | MW106962 |
|  | A/chicken/Xinjiang/016/2017 | H9N2 | MW106954 |
|  | A/environment/Xinjiang/017/2017 | H9N2 | MW099793 |
|  | A/chicken/Xinjiang/018/2017 | H9N2 | MW099794 |
|  | A/chicken/Xinjiang/019/2017 | H9N2 | MW099795 |
|  | A/chicken/Xinjiang/020/2017 | H9N2 | MW099796 |
|  | A/chicken/Xinjiang/021/2017 | H9N2 | MW099797 |
|  | A/chicken/Xinjiang/022/2018 | H9N2 | MW099951 |
|  | A/chicken/Xinjiang/023/2018 | H9N2 | MW099952 |
|  | A/chicken/Xinjiang/024/2018 | H9N2 | MW106978 |
|  | A/chicken/Xinjiang/025/2018 | H9N2 | MW099953 |
|  | A/chicken/Xinjiang/026/2018 | H9N2 | MW099954 |
|  | A/chicken/Xinjiang/027/2018 | H9N2 | MW099955 |
|  | A/chicken/Xinjiang/028/2018 | H9N2 | MW099956 |
|  | A/duck/Xinjiang/029/2018 | H9N2 | MW099957 |
|  | A/chicken/Xinjiang/030/2018 | H9N2 | MW099958 |

| PB2 | Strain name | Subtype | Isolate_ID |
| --- | --- | --- | --- |
| GISAID | A/Beijing/28244/2017 | H7N9 | EPI_ISL_285009 |
|  | A/Fujian-Sanyuan/2881/2019 | H9N2 | EPI_ISL_407978 |
|  | A/chicken/China/a45/2017 | H9N2 | EPI_ISL_301896 |
|  | A/Henan/27454/2017 | H7N9 | EPI_ISL_285176 |
|  | A/Anhui/13423/2017 | H7N9 | EPI_ISL_258012 |
|  | A/Henan/32240/2017 | H7N9 | EPI_ISL_285179 |
|  | A/Beijing/27869/2017 | H7N9 | EPI_ISL_285007 |
|  | A/Hunan/42088/2017 | H9N2 | EPI_ISL_337279 |
|  | A/Environment/Hunan/28221/2016 | H9N2 | EPI_ISL_283838 |
|  | A/Environment/Hunan/44973/2015 | H9N2 | EPI_ISL_283849 |
|  | A/Guangdong/60061/2016 | H7N9 | EPI_ISL_242888 |
|  | A/Environment/Guangdong/16725/2017 | H9N2 | EPI_ISL_283959 |
|  | A/Environment/Guangzhou/4188/2016 | H7N9 | EPI_ISL_273947 |
|  | A/Guangdong/17SF036/2017 | H7N9 | EPI_ISL_285133 |
|  | A/Environment/Guangzhou/5258-5259/2017 | H7N9 | EPI_ISL_273953 |
|  | A/Guangdong/Zhuhai/20SF8034/2020 | H9N2 | EPI_ISL_447901 |
|  | A/Guangdong/18SF064/2018 | H9N2 | EPI_ISL_345234 |
|  | A/Guangdong/18SF003/2018 | H9N2 | EPI_ISL_337277 |
|  | A/Anhui/42444/2015 | H7N9 | EPI_ISL_284991 |
|  | A/Environment/Jiangxi/47554/2015 | H9N2 | EPI_ISL_283872 |
|  | A/Environment/Fujian/41640/2015 | H7N9 | EPI_ISL_277446 |
|  | A/Environment/Yunnan/01451/2015 | H9N2 | EPI_ISL_283941 |
|  | A/Environment/Guangxi/35160/2016 | H9N2 | EPI_ISL_283808 |
|  | A/Environment/Guangdong/35200/2016 | H9N2 | EPI_ISL_283783 |
|  | A/Environment/Hunan/46747/2015 | H9N2 | EPI_ISL_283855 |
|  | A/Anhui/26836/2016 | H7N9 | EPI_ISL_284982 |
|  | A/Environment/Ningxia/58578/2016 | H9N2 | EPI_ISL_283882 |
|  | A/chicken/ShanDong/210WZ/2017 | H9N2 | EPI_ISL_281315 |
|  | A/Duck/China/F908/2015 | H9N2 | EPI_ISL_501327 |
|  | A/Environment/Fujian/05314/2015 | H9N2 | EPI_ISL_283744 |
|  | A/Environment/ Qingdao/29/2017 | H9N2 | EPI_ISL_283911 |
|  | A/Henan/17867/2017 | H7N9 | EPI_ISL_285173 |
|  | A/Sichuan/24986/2017 | H7N9 | EPI_ISL_285288 |
|  | A/Guangdong/17SF009/2017 | H7N9 | EPI_ISL_285125 |
|  | A/Environment/Anhui/54850/2016 | H9N2 | EPI_ISL_283717 |
|  | A/Environment/Chongqing/38212/2016 | H9N2 | EPI_ISL_283723 |
|  | A/Environment/Yunnan/60642/2016 | H9N2 | EPI_ISL_283952 |
|  | A/Environment/Shandong/227150/2017 | H9N2 | EPI_ISL_283901 |
|  | A/Environment/Gansu/52441/2016 | H9N2 | EPI_ISL_283766 |
|  | A/duck/Shantou/7488/2004 | H9N2 | EPI_ISL_7405 |
|  | A/Chicken/Korea/38349-p96323/96 | H9N2 | EPI_ISL_1274 |
|  | A/Chicken/Korea/25232-96006/96 | H9N2 | EPI_ISL_1275 |
|  | A/Quail/Hong-Kong/G1/97 | H9N2 | EPI_ISL_1268 |
|  | A/chicken/Guangdong/6/97 | H9N2 | EPI_ISL_8500 |
|  | A/Duck/Hong-Kong/Y439/97 | H9N2 | EPI_ISL_1267 |
|  | A/Chicken/Beijing/1/94 | H9N2 | EPI_ISL_1270 |
|  | A/Duck/Hong Kong/Y280/97 | H9N2 | EPI_ISL_1266 |
| NCBI | A/Chicken/Shandong/6/96 | H9N2 | DQ064565 |
|  | A/turkey/Wisconsin/1/1966 | H9N2 | CY014670 |
|  | A/chicken/ShangDong/16587/2018 | H9N2 | MK367651 |
|  | A/chicken/Anhui/LH99/2017 | H9N2 | MH489448 |
|  | A/chicken/Shandong/WF75/2017 | H9N2 | MH489472 |
|  | A/chicken/Shanghai/1106-58/2017 | H9N2 | MG984047 |
|  | A/chicken/Shandong/3424/2016 | H9N2 | MH667569 |
|  | A/Falco-tinnunculus/Tianjin/04/2017 | H9N2 | MH114039 |
|  | A/Athene-noctua/Tianjin/Y14/2017 | H9N2 | MH114055 |
|  | A/environment/Guangdong/S12412/2017 | H7N9 | MH209536 |
|  | A/chicken/China/355/2017 | H9N2 | MN385376 |
|  | A/Guangdong/SP440/2017 | H7N9 | KY643838 |
|  | A/Guangdong/HP001/2017 | H7N9 | KY643837 |
|  | A/chicken/China/93/2017 | H9N2 | MN385406 |
|  | A/chicken/China/G1301PB216GD/2016 | H9N2 | MK326885 |
|  | A/duck/China/D4/2018 | H9N2 | MN384773 |
|  | A/chicken/China/728/2017 | H9N2 | MN385393 |
|  | A/chicken/Qingyuan/zd201602/2016 | H9N2 | MK250030 |
|  | A/chicken/Vietnam/HU9-567/2018 | H9N2 | LC497158 |
|  | A/duck/Wenzhou/YHQL64/2014 | H9N2 | KU143583 |
|  | A/chicken/Yuhuan/YH15/2016 | H9N2 | KY415893 |
|  | A/chicken/Shanghai/15/2015 | H9N2 | KU720462 |
|  | A/enviroment/Hubei/S0139/2016 | H9N2 | MN647490 |
|  | A/duck/Japan/AQ-HE5/2015 | H9N2 | LC208502 |
|  | A/chicken/Jiangsu/S1045/2016 | H7N9 | MF630234 |
|  | A/chicken/China/333/2017 | H9N2 | MH588117 |
|  | A/chicken/Zhejiang/HJ/2007 | H9N2 | FJ581429 |
|  | A/Chicken/Hong-Kong/G9/97 | H9N2 | KF188370 |
|  | A/Chicken/Shanghai/F/98 | H9N2 | AY253750 |
|  | A/chicken/Xinjiang/01/2017 | H9N2 | MW094654 |
|  | A/chicken/Xinjiang/02/2017 | H9N2 | MW094655 |
|  | A/duck/Xinjiang/03/2017 | H9N2 | MW094656 |
|  | A/duck/Xinjiang/04/2017 | H9N2 | MW094657 |
|  | A/chicken/Xinjiang/05/2017 | H9N2 | MW106989 |
|  | A/chicken/Xinjiang/06/2017 | H9N2 | MW106997 |
|  | A/chicken/Xinjiang/07/2017 | H9N2 | MW094659 |
|  | A/chicken/Xinjiang/08/2017 | H9N2 | MW094660 |
|  | A/chicken/Xinjiang/09/2017 | H9N2 | MW094661 |
|  | A/chicken/Xinjiang/010/2017 | H9N2 | MW094662 |
|  | A/chicken/Xinjiang/011/2017 | H9N2 | MW106941 |
|  | A/chicken/Xinjiang/012/2017 | H9N2 | MW094663 |
|  | A/chicken/Xinjiang/013/2017 | H9N2 | MW094664 |
|  | A/chicken/Xinjiang/014/2017 | H9N2 | MW094665 |
|  | A/chicken/Xinjiang/015/2017 | H9N2 | MW106957 |
|  | A/chicken/Xinjiang/016/2017 | H9N2 | MW106949 |
|  | A/environment/Xinjiang/017/2017 | H9N2 | MW094666 |
|  | A/chicken/Xinjiang/018/2017 | H9N2 | MW094667 |
|  | A/chicken/Xinjiang/019/2017 | H9N2 | MW094668 |
|  | A/chicken/Xinjiang/020/2017 | H9N2 | MW094669 |
|  | A/chicken/Xinjiang/021/2017 | H9N2 | MW094670 |
|  | A/chicken/Xinjiang/022/2018 | H9N2 | MW094820 |
|  | A/chicken/Xinjiang/023/2018 | H9N2 | MW094821 |
|  | A/chicken/Xinjiang/024/2018 | H9N2 | MW106973 |
|  | A/chicken/Xinjiang/025/2018 | H9N2 | MW094822 |
|  | A/chicken/Xinjiang/026/2018 | H9N2 | MW094823 |
|  | A/chicken/Xinjiang/027/2018 | H9N2 | MW094824 |
|  | A/chicken/Xinjiang/028/2018 | H9N2 | MW094825 |
|  | A/duck/Xinjiang/029/2018 | H9N2 | MW094826 |
|  | A/chicken/Xinjiang/030/2018 | H9N2 | MW094827 |

| PB1 | Strain name | Subtype | Isolate_ID |
| --- | --- | --- | --- |
| GISAID | A/Jiangxi/10666/2017 | H7N9 | EPI_ISL_285235 |
|  | A/Environment/Hunan/12811/2017 | H9N2 | EPI_ISL_283832 |
|  | A/Environment/Shandong-Rizhao/09/2017 | H9N2 | EPI_ISL_283912 |
|  | A/Shandong-Qingdao/03/2017 | H7N9 | EPI_ISL_285266 |
|  | A/Environment/Guangdong/16725/2017 | H9N2 | EPI_ISL_283959 |
|  | A/Guangdong/18SF003/2018 | H9N2 | EPI_ISL_337277 |
|  | A/chicken/Guangdong/DGCPLB032/2016 | H7N9 | EPI_ISL_259747 |
|  | A/Chicken/Guangdong/DG16800/2016 | H7N9 | EPI_ISL_249154 |
|  | A/Shenzhen/Th004/2017 | H7N9 | EPI_ISL_250314 |
|  | A/Hong Kong/125/2017 | H7N9 | EPI_ISL_259269 |
|  | A/Environment/Guangdong/60345/2016 | H9N2 | EPI_ISL_283796 |
|  | A/Environment/Guangdong/07104/2017 | H7N9 | EPI_ISL_285026 |
|  | A/Sichuan/24980/2017 | H7N9 | EPI_ISL_285282 |
|  | A/Environment/Inner-Mongolia/28670/2017 | H7N9 | EPI_ISL_285062 |
|  | A/Fujian-Sanyuan/2881/2019 | H9N2 | EPI_ISL_407978 |
|  | A/Hunan/09917/2017 | H7N9 | EPI_ISL_285203 |
|  | A/Shanghai/PD04/2015 | H7N9 | EPI_ISL_221671 |
|  | A/chicken/China/F344/2015 | H9N2 | EPI_ISL_501324 |
|  | A/Environment/Gansu/52441/2016 | H9N2 | EPI_ISL_283766 |
|  | A/Beijing/27870/2017 | H7N9 | EPI_ISL_285008 |
|  | A/Hebei/24991/2017 | H7N9 | EPI_ISL_285153 |
|  | A/Sichuan/27873/2017 | H7N9 | EPI_ISL_285297 |
|  | A/Environment/Chongqing/38212/2016 | H9N2 | EPI_ISL_283723 |
|  | A/Xinjiang/04062/2018 | H7N9 | EPI_ISL_325939 |
|  | A/chicken/Hangzhou/174/2013 | H9N2 | EPI_ISL_194993 |
|  | A/Chicken/Guangdong/DG16437/2016 | H7N9 | EPI_ISL_249140 |
|  | A/Anhui/40095/2015 | H7N9 | EPI_ISL_192472 |
|  | A/environment/Hunan/YYGK388/2015 | H9N2 | EPI_ISL_199111 |
|  | A/environment/sichuan/322077/2015 | H9N2 | EPI_ISL_283924 |
|  | A/Beijing/39450/2016 | H7N9 | EPI_ISL_285014 |
|  | A/Sichuan/08606/2017 | H7N9 | EPI_ISL_285272 |
|  | A/Environment/Hunan/12613/2017 | H9N2 | EPI_ISL_283831 |
|  | A/chicken/Zhejiang/HJ/2007 | H9N2 | EPI_ISL_63471 |
|  | A/Chicken/Shanghai/F/98 | H9N2 | EPI_ISL_68579 |
|  | A/Duck/Hong Kong/Y439/97 | H9N2 | EPI_ISL_1267 |
|  | A/duck/Hong Kong/784/1979 | H9N2 | EPI_ISL_1273 |
|  | A/Turkey/California/189/66 | H9N2 | EPI_ISL_1280 |
|  | A/turkey/Wisconsin/1/1966 | H9N2 | EPI_ISL_70131 |
|  | A/Chicken/Beijing/1/94 | H9N2 | EPI_ISL_1270 |
|  | A/Duck/Hong Kong/Y280/97 | H9N2 | EPI_ISL_1266 |
|  | A/Swine/Hong Kong/9/98 | H9N2 | EPI_ISL_142 |
|  | A/Chicken/Korea/38349-p96323/96 | H9N2 | EPI_ISL_1274 |
|  | A/Chicken/Korea/25232-96006/96 | H9N2 | EPI_ISL_1275 |
|  | A/Pigeon/Hong Kong/Y233/97 | H9N2 | EPI_ISL_1265 |
|  | A/Hong Kong/1073/99 | H9N2 | EPI_ISL_140 |
|  | A/Quail/Hong Kong/G1/97 | H9N2 | EPI_ISL_1268 |
|  | A/chicken/Heilongjiang/35/00 | H9N2 | EPI_ISL_8504 |
|  | A/Guangzhou/333/99 | H9N2 | EPI_ISL_3046 |
| NCBI | A/chicken/China/G1639PB116GD/2016 | H9N2 | MK326878 |
|  | A/duck/Ganzhou/GZ188/2016 | H9N2 | KY415860 |
|  | A/chicken/China/237/2017 | H9N2 | MN385369 |
|  | A/chicken/Hubei/01/2015 | H9N2 | MF568509 |
|  | A/chicken/Shandong/3442/2017 | H9N2 | MH667599 |
|  | A/Falco tinnunculus/Tianjin/04/2017 | H9N2 | MH114040 |
|  | A/chicken/ShanDong/210WZ/2017 | H9N2 | MF795008 |
|  | A/chicken/China/241/2017 | H9N2 | MH588105 |
|  | A/environment/Zhongshan/ZS201601/2016 | H9N2 | KX783273 |
|  | A/chicken/Shandong/1834/2019 | H9N2 | MN765113 |
|  | A/chicken/Guangdong/Q26/2017 | H7N9 | MF280185 |
|  | A/chicken/China/355/2017 | H9N2 | MN385377 |
|  | A/chicken/Zhejiang/221/2016 | H9N2 | KY056277 |
|  | A/chicken/Jiangsu/SD012/2015 | H7N9 | MF630275 |
|  | A/chicken/Shanghai/1127-30/2017 | H9N2 | MG984043 |
|  | A/chicken/Xuancheng/01/2018 | H9N2 | MK138613 |
|  | A/chicken/Shandong/WF75/2017 | H9N2 | MH489473 |
|  | A/Kunming/KMCDC-YHY/2017 | H7N9 | MG366899 |
|  | A/chicken/Zhejiang/SIC40/2015 | H9N2 | KX598594 |
|  | A/chicken/Shanghai/06/2015 | H9N2 | MK053860 |
|  | A/chicken/Wenzhou/HATSLG01/2015 | H7N9 | KU143550 |
|  | A/duck/Wenzhou/YHQL64/2014 | H9N2 | KU143540 |
|  | A/chicken/Shanghai/S4100/2015 | H7N9 | MF630331 |
|  | A/chicken/Shandong/yt0106/2012 | H9N2 | KM609803 |
|  | A/chicken/Zhejiang/SC324/2013 | H9N2 | KM113059 |
|  | A/chicken/Jiangxi/13212/2014(Mixed) | H9N2 | KP287519 |
|  | A/chicken/Shandong/SIC34/2014 | H9N2 | KX598586 |
|  | A/duck/Japan/AQ-HE5/2015 | H9N2 | LC208503 |
|  | A/Duck/Hong Kong/702/79 | H9N2 | CY031266 |
|  | A/Chicken/Hong Kong/G9/97 | H9N2 | KF188370 |
|  | A/chicken/Xinjiang/01/2017 | H9N2 | MW095595 |
|  | A/chicken/Xinjiang/02/2017 | H9N2 | MW095596 |
|  | A/duck/Xinjiang/03/2017 | H9N2 | MW095597 |
|  | A/duck/Xinjiang/04/2017 | H9N2 | MW095598 |
|  | A/chicken/Xinjiang/05/2017 | H9N2 | MW106990 |
|  | A/chicken/Xinjiang/06/2017 | H9N2 | MW106998 |
|  | A/chicken/Xinjiang/07/2017 | H9N2 | MW095600 |
|  | A/chicken/Xinjiang/08/2017 | H9N2 | MW095601 |
|  | A/chicken/Xinjiang/09/2017 | H9N2 | MW095602 |
|  | A/chicken/Xinjiang/010/2017 | H9N2 | MW095603 |
|  | A/chicken/Xinjiang/011/2017 | H9N2 | MW106942 |
|  | A/chicken/Xinjiang/012/2017 | H9N2 | MW095604 |
|  | A/chicken/Xinjiang/013/2017 | H9N2 | MW095605 |
|  | A/chicken/Xinjiang/014/2017 | H9N2 | MW095606 |
|  | A/chicken/Xinjiang/015/2017 | H9N2 | MW106958 |
|  | A/chicken/Xinjiang/016/2017 | H9N2 | MW106950 |
|  | A/environment/Xinjiang/017/2017 | H9N2 | MW095607 |
|  | A/chicken/Xinjiang/018/2017 | H9N2 | MW095608 |
|  | A/chicken/Xinjiang/019/2017 | H9N2 | MW095609 |
|  | A/chicken/Xinjiang/020/2017 | H9N2 | MW095610 |
|  | A/chicken/Xinjiang/021/2017 | H9N2 | MW095611 |
|  | A/chicken/Xinjiang/022/2018 | H9N2 | MW095765 |
|  | A/chicken/Xinjiang/023/2018 | H9N2 | MW095766 |
|  | A/chicken/Xinjiang/024/2018 | H9N2 | MW106974 |
|  | A/chicken/Xinjiang/025/2018 | H9N2 | MW095767 |
|  | A/chicken/Xinjiang/026/2018 | H9N2 | MW095768 |
|  | A/chicken/Xinjiang/027/2018 | H9N2 | MW095769 |
|  | A/chicken/Xinjiang/028/2018 | H9N2 | MW095770 |
|  | A/duck/Xinjiang/029/2018 | H9N2 | MW095771 |
|  | A/chicken/Xinjiang/030/2018 | H9N2 | MW095772 |

| PA | Strain name | Subtype | Isolate_ID |
| --- | --- | --- | --- |
| GISAID | A/swine/Shandong/TA009/2019 | H9N2 | EPI_ISL_503942 |
|  | A/Hunan/42088/2017 | H9N2 | EPI_ISL_337279 |
|  | A/Anhui-Lujiang/39/2018 | H9N2 | EPI_ISL_330737 |
|  | A/Environment/Shandong/06127/2015 | H9N2 | EPI_ISL_283892 |
|  | A/chicken/China/E1974/2014 | H9N2 | EPI_ISL_501301 |
|  | A/Environment/Anhui/42489/2015 | H9N2 | EPI_ISL_283713 |
|  | A/Environment/Guangdong/35257/2016 | H9N2 | EPI_ISL_283784 |
|  | A/chicken/China/F2170/2015 | H9N2 | EPI_ISL_501339 |
|  | A/Environment/Jiangsu/03758/2015 | H7N9 | EPI_ISL_285063 |
|  | A/chicken/Wuxi/7144/2015 | H7N9 | EPI_ISL_277046 |
|  | A/Environment/Hunan/08069/2017 | H7N9 | EPI_ISL_285049 |
|  | A/Jiangsu/08188/2016 | H7N9 | EPI_ISL_285378 |
|  | A/Hunan/06947/2017 | H7N9 | EPI_ISL_285189 |
|  | A/Environment/Jiangsu/12052/2016 | H7N9 | EPI_ISL_285069 |
|  | A/Shenzhen/TH003/2016 | H5N6 | EPI_ISL_250313 |
|  | A/Guangdong/17SF064/2017 | H7N9 | EPI_ISL_267764 |
|  | A/environment/Guangdong/SZBA-E1/2017 | H7N9 | EPI_ISL_259759 |
|  | A/Guangdong/GZ8H001/2017 | H7N9 | EPI_ISL_327792 |
|  | A/Guangdong/HP001/2017 | H7N9 | EPI_ISL_256108\| |
|  | A/Guangdong/18SF064/2018 | H9N2 | EPI_ISL_345234 |
|  | A/Guangxi-Xiangshan/11522/2018 | H9N2 | EPI_ISL_345235 |
|  | A/Chicken/Shanghai/F/98 | H9N2 | EPI_ISL_68579 |
|  | A/chicken/Zhejiang/HJ/2007 | H9N2 | EPI_ISL_63471 |
|  | A/Chicken/Hong Kong/G9/97 | H9N2 | EPI_ISL_1263 |
|  | A/Chicken/Shandong/6/96 | H9N2 | EPI_ISL_2240 |
|  | A/Duck/Hong Kong/Y280/97 | H9N2 | EPI_ISL_1266 |
|  | A/Chicken/Beijing/1/94 | H9N2 | EPI_ISL_1270 |
|  | A/chicken/Heilongjiang/35/00 | H9N2 | EPI_ISL_8504 |
|  | A/Guangzhou/333/99 | H9N2 | EPI_ISL_3046 |
|  | A/Quail/Hong Kong/G1/97 | H9N2 | EPI_ISL_1268 |
|  | A/Hong Kong/1073/99 | H9N2 | EPI_ISL_140 |
|  | A/duck/Hong Kong/784/1979 | H9N2 | EPI_ISL_1273 |
|  | A/Duck/Hong Kong/Y439/97 | H9N2 | EPI_ISL_1267 |
|  | A/Chicken/Korea/38349-p96323/96 | H9N2 | EPI_ISL_1274 |
|  | A/Chicken/Korea/25232-96006/96 | H9N2 | EPI_ISL_1275 |
|  | A/Turkey/California/189/66 | H9N2 | EPI_ISL_1280 |
|  | A/turkey/Wisconsin/1/1966 | H9N2 | EPI_ISL_70131 |
| NCBI | A/environment-air/Kunshan/NIOSH-BL20/2018 | H9N2 | MN606220 |
|  | A/chicken/Shandong/WF75/2017 | H9N2 | MH489474 |
|  | A/chicken/ShangDong/16587/2018 | H9N2 | MK367653 |
|  | A/chicken/Anhui/LH99/2017 | H9N2 | MH489450 |
|  | A/chicken/Shandong/3442/2017 | H9N2 | MH667600 |
|  | A/duck/China/D4/2018 | H9N2 | MN384775 |
|  | A/chicken/China/1104/2019 | H9N2 | MN918148 |
|  | A/Falco tinnunculus/Tianjin/04/2017 | H9N2 | MH114041 |
|  | A/chicken/China/348/2018 | H9N2 | MH588127 |
|  | A/chicken/Anhui/AH480/2017 | H9N2 | MH489506 |
|  | A/chicken/Shanghai/11/2018 | H9N2 | MK053875 |
|  | A/chicken/China/1642.3/2018 | H9N2 | MK367621 |
|  | A/chicken/Xuzhou/XZ270/2016 | H9N2 | MG063474 |
|  | A/wild birds/Hubei/45/2014 | H9N2 | MH991754 |
|  | A/chicken/Hubei/01/2015 | H9N2 | MF568510 |
|  | A/chicken/ShanDong/217YY/2017 | H9N2 | MF795041 |
|  | A/chicken/Shanghai/S4100/2015 | H7N9 | MF630332 |
|  | A/chicken/Jiangsu/TM210/2016 | H9N2 | MN135908 |
|  | A/chicken/Jiangxi/33583/2013 (Mixed) | H9N2 | KP416670 |
|  | A/chicken/China/G1728PA16GD/2016 | H9N2 | MK326859 |
|  | A/environment/Hunan/18498/2014 | H9N2 | KT356743 |
|  | A/chicken/Jiangxi/31872/2013 | H9N2 | KP285436 |
|  | A/chicken/China/G1737PA16GD/2016 | H9N2 | MK326862 |
|  | A/chicken/Shandong/qd0427/2012 | H9N2 | KM609776 |
|  | A/chicken/Guangdong/SD1433/2016 | H7N9 | MF630156 |
|  | A/chicken/Qingyuan/zd201602/2016 | H9N2 | MK250032 |
|  | A/chicken/China/93/2017 | H9N2 | MN385408 |
|  | A/Duck/Hong Kong/702/79 | H9N2 | CY031266 |
|  | A/chicken/Xinjiang/01/2017 | H9N2 | MW096521 |
|  | A/chicken/Xinjiang/02/2017 | H9N2 | MW096522 |
|  | A/duck/Xinjiang/03/2017 | H9N2 | MW096523 |
|  | A/duck/Xinjiang/04/2017 | H9N2 | MW096524 |
|  | A/chicken/Xinjiang/05/2017 | H9N2 | MW106991 |
|  | A/chicken/Xinjiang/06/2017 | H9N2 | MW106999 |
|  | A/chicken/Xinjiang/07/2017 | H9N2 | MW096526 |
|  | A/chicken/Xinjiang/08/2017 | H9N2 | MW096527 |
|  | A/chicken/Xinjiang/09/2017 | H9N2 | MW096528 |
|  | A/chicken/Xinjiang/010/2017 | H9N2 | MW096529 |
|  | A/chicken/Xinjiang/011/2017 | H9N2 | MW106943 |
|  | A/chicken/Xinjiang/012/2017 | H9N2 | MW096530 |
|  | A/chicken/Xinjiang/013/2017 | H9N2 | MW096531 |
|  | A/chicken/Xinjiang/014/2017 | H9N2 | MW096532 |
|  | A/chicken/Xinjiang/015/2017 | H9N2 | MW106959 |
|  | A/chicken/Xinjiang/016/2017 | H9N2 | MW106951 |
|  | A/environment/Xinjiang/017/2017 | H9N2 | MW096533 |
|  | A/chicken/Xinjiang/018/2017 | H9N2 | MW096534 |
|  | A/chicken/Xinjiang/019/2017 | H9N2 | MW096535 |
|  | A/chicken/Xinjiang/020/2017 | H9N2 | MW096536 |
|  | A/chicken/Xinjiang/021/2017 | H9N2 | MW096537 |
|  | A/chicken/Xinjiang/022/2018 | H9N2 | MW096685 |
|  | A/chicken/Xinjiang/023/2018 | H9N2 | MW096686 |
|  | A/chicken/Xinjiang/024/2018 | H9N2 | MW106975 |
|  | A/chicken/Xinjiang/025/2018 | H9N2 | MW096687 |
|  | A/chicken/Xinjiang/026/2018 | H9N2 | MW096688 |
|  | A/chicken/Xinjiang/027/2018 | H9N2 | MW096689 |
|  | A/chicken/Xinjiang/028/2018 | H9N2 | MW096690 |
|  | A/duck/Xinjiang/029/2018 | H9N2 | MW096691 |
|  | A/chicken/Xinjiang/030/2018 | H9N2 | MW096692 |

| NP | Strain name | Subtype | Isolate_ID |
| --- | --- | --- | --- |
| GISAID | A/Environment/Shandong-Rizhao/35/2017 | H9N2 | EPI_ISL_283915 |
|  | A/Environment/Yunnan/60642/2016 | H9N2 | EPI_ISL_283952 |
|  | A/chicken/China/333/2017 | H9N2 | EPI_ISL_328163 |
|  | A/Environment/Shandong/227150/2017 | H9N2 | EPI_ISL_283901 |
|  | A/Environment/Chongqing/38212/2016 | H9N2 | EPI_ISL_283723 |
|  | A/chicken/Shandong/k147/2017 | H9N2 | EPI_ISL_505072 |
|  | A/chicken/Jiangsu/JS11/2016 | H9N2 | EPI_ISL_305589 |
|  | A/Environment/Zhejiang/54142/2016 | H9N2 | EPI_ISL_283955 |
|  | A/Hunan/09922/2017 | H7N9 | EPI_ISL_285213 |
|  | A/Environment/Gansu/52450/2016 | H9N2 | EPI_ISL_283767 |
|  | A/duck/Ganzhou/GZ188/2016 | H9N2 | EPI_ISL_252836 |
|  | A/chicken/Shanghai/15/2015 | H9N2 | EPI_ISL_215584 |
|  | A/chicken/Xuancheng/01/2018 | H9N2 | EPI_ISL_379404 |
|  | A/duck/Wenzhou/YHQL64/2014 | H9N2 | EPI_ISL_205130 |
|  | A/Environment/Xinjiang/39018/2015 | H9N2 | EPI_ISL_329998 |
|  | A/chicken/Xuzhou/XZ270/2016 | H9N2 | EPI_ISL_284665 |
|  | A/Hunan/34179/2018 | H9N2 | EPI_ISL_345236 |
|  | A/chicken/Shanghai/06/2018 | H9N2 | EPI_ISL_379840 |
|  | A/Environment/Gansu/55642/2016 | H9N2 | EPI_ISL_283770 |
|  | A/chicken/China/71/2019 | H9N2 | EPI_ISL_381687 |
|  | A/chicken/China/1103/2019 | H9N2 | EPI_ISL_505806 |
|  | A/chicken/Shandong/3424/2016 | H9N2 | EPI_ISL_505068 |
|  | A/chicken/Primorsky Krai/1771/2018 | H9N2 | EPI_ISL_336927 |
|  | A/chicken/Primorsky Krai/03/2018 | H9N2 | EPI_ISL_321315 |
|  | A/chicken/China/G1737NP16GD/2016 | H9N2 | EPI_ISL_399108 |
|  | A/Guangdong/18SF064/2018 | H9N2 | EPI_ISL_345234 |
|  | A/chicken/Guangdong/SZBJ0011-O/2017 | H7N9 | EPI_ISL_259758 |
|  | A/Guangdong/17SF061/2017 | H7N9 | EPI_ISL_285136 |
|  | A/Guangdong/17SF042/2017 | H7N9 | EPI_ISL_285134 |
|  | A/Guangxi/2/2017 | H7N9 | EPI_ISL_268515 |
|  | A/chicken/Hunan/YueYang0501/2015 | H9N2 | EPI_ISL_200631 |
|  | A/duck/Hunan/YYGK250-P/2014 | H9N2 | EPI_ISL_198790 |
|  | A/Environment/Shandong/02186/2016 | H9N2 | EPI_ISL_283887 |
|  | A/environment/Jiangxi/NCJD0011D/2015(Mixed) | H9N6 | EPI_ISL_200403 |
|  | A/chicken/Hubei/2014 | H9N2 | EPI_ISL_191490 |
|  | A/Environment/Hunan/35089/2016 | H9N2 | EPI_ISL_283846 |
|  | A/chicken/Viet Nam/LS-0119/2013 | H9N2 | EPI_ISL_387686 |
|  | A/chicken/Shandong/SIC34/2014 | H9N2 | EPI_ISL_234453 |
|  | A/chicken/Ganzhou/GZ86/2016 | H9N2 | EPI_ISL_252835 |
|  | A/chicken/Jiangxi/NCDZT0055-O/2015 | H9N6 | EPI_ISL_198983 |
|  | A/duck/Hubei/WHWTZ0108-P/2015(mixed) | H7N9 | EPI_ISL_200422 |
|  | A/duck/Hunan/YYGK0045-P/2014 | H9N6 | EPI_ISL_200388 |
|  | A/Beijing/39450/2016 | H7N9 | EPI_ISL_285014 |
|  | A/Environment/Jiangsu/12054/2016 | H9N2 | EPI_ISL_283863 |
|  | A/Jiangsu/08207/2016 | H7N9 | EPI_ISL_285407 |
|  | A/environment/Hunan/S40858/2015 | H7N9 | EPI_ISL_283553 |
|  | A/environment/sichuan/322078/2015 | H9N2 | EPI_ISL_283925 |
|  | A/chicken/Wuxi/6082/2015 | H9N2 | EPI_ISL_277064 |
|  | A/Chicken/Guangdong/DG16800/2016 | H7N9 | EPI_ISL_249154 |
|  | A/Environment/Hunan/12613/2017 | H9N2 | EPI_ISL_283831 |
|  | A/Environment/Jiangxi/47554/2015 | H9N2 | EPI_ISL_283872 |
|  | A/chicken/Zhejiang/HJ/2007 | H9N2 | EPI_ISL_63471 |
|  | A/Chicken/Shanghai/F/98 | H9N2 | EPI_ISL_68579 |
|  | A/Quail/Hong Kong/G1/97 | H9N2 | EPI_ISL_1268 |
|  | A/Hong Kong/1073/99 | H9N2 | EPI_ISL_140 |
|  | A/Duck/Hong Kong/Y439/97 | H9N2 | EPI_ISL_1267 |
|  | A/Chicken/Korea/38349-p96323/96 | H9N2 | EPI_ISL_1274 |
|  | A/Chicken/Korea/25232-96006/96 | H9N2 | EPI_ISL_1275 |
|  | A/chicken/Heilongjiang/35/00 | H9N2 | EPI_ISL_8504 |
|  | A/turkey/Wisconsin/1/1966 | H9N2 | EPI_ISL_70131 |
|  | A/Turkey/California/189/66 | H9N2 | EPI_ISL_1280 |
|  | A/Duck/Hong Kong/702/79 | H9N2 | EPI_ISL_139 |
|  | A/Chicken/Beijing/1/94 | H9N2 | EPI_ISL_1270 |
|  | A/Guangzhou/333/99 | H9N2 | EPI_ISL_3046 |
|  | A/Chicken/Shandong/6/96 | H9N2 | EPI_ISL_2240 |
|  | A/Swine/Hong Kong/9/98 | H9N2 | EPI_ISL_142 |
|  | A/Chicken/Hong Kong/G9/97 | H9N2 | EPI_ISL_146698 |
|  | A/Duck/Hong Kong/Y280/97 | H9N2 | EPI_ISL_1266 |
| NCBI | A/Accipiter-gentilis-schvedowi/Tianjin/22/2017 | H9N2 | MH114051 |
|  | A/environment-air/Kunshan/NIOSH-BL53/2018 | H9N2 | MN607192 |
|  | A/chicken/China/93/2017 | H9N2 | MN385410 |
|  | A/chicken/Xinjiang/01/2017 | H9N2 | MW098859 |
|  | A/chicken/Xinjiang/02/2017 | H9N2 | MW098860 |
|  | A/duck/Xinjiang/03/2017 | H9N2 | MW098861 |
|  | A/duck/Xinjiang/04/2017 | H9N2 | MW098862 |
|  | A/chicken/Xinjiang/05/2017 | H9N2 | MW106993 |
|  | A/chicken/Xinjiang/06/2017 | H9N2 | MW107001 |
|  | A/chicken/Xinjiang/07/2017 | H9N2 | MW098864 |
|  | A/chicken/Xinjiang/08/2017 | H9N2 | MW098865 |
|  | A/chicken/Xinjiang/09/2017 | H9N2 | MW098866 |
|  | A/chicken/Xinjiang/010/2017 | H9N2 | MW098867 |
|  | A/chicken/Xinjiang/011/2017 | H9N2 | MW106945 |
|  | A/chicken/Xinjiang/012/2017 | H9N2 | MW098868 |
|  | A/chicken/Xinjiang/013/2017 | H9N2 | MW098869 |
|  | A/chicken/Xinjiang/014/2017 | H9N2 | MW098870 |
|  | A/chicken/Xinjiang/015/2017 | H9N2 | MW106961 |
|  | A/chicken/Xinjiang/016/2017 | H9N2 | MW106953 |
|  | A/environment/Xinjiang/017/2017 | H9N2 | MW098871 |
|  | A/chicken/Xinjiang/018/2017 | H9N2 | MW098872 |
|  | A/chicken/Xinjiang/019/2017 | H9N2 | MW098873 |
|  | A/chicken/Xinjiang/020/2017 | H9N2 | MW098874 |
|  | A/chicken/Xinjiang/021/2017 | H9N2 | MW098875 |
|  | A/chicken/Xinjiang/022/2018 | H9N2 | MW099028 |
|  | A/chicken/Xinjiang/023/2018 | H9N2 | MW099029 |
|  | A/chicken/Xinjiang/024/2018 | H9N2 | MW106977 |
|  | A/chicken/Xinjiang/025/2018 | H9N2 | MW099030 |
|  | A/chicken/Xinjiang/026/2018 | H9N2 | MW099031 |
|  | A/chicken/Xinjiang/027/2018 | H9N2 | MW099032 |
|  | A/chicken/Xinjiang/028/2018 | H9N2 | MW099033 |
|  | A/duck/Xinjiang/029/2018 | H9N2 | MW099034 |
|  | A/chicken/Xinjiang/030/2018 | H9N2 | MW099035 |

| MP | Strain name | Subtype | Isolate_ID |
| --- | --- | --- | --- |
| GISAID | A/chicken/Jiangsu/YZLH37/2017 | H7N9 | EPI_ISL_378712 |
|  | A/Environment/Guangdong/47405/2016 | H9N2 | EPI_ISL_283793 |
|  | A/Environment/Jiangsu/06662/2016 | H7N9 | EPI_ISL_285065 |
|  | A/Jiangsu/60463/2016 | H7N9 | EPI_ISL_242875 |
|  | A/Jiangsu/08190/2017 | H7N9 | EPI_ISL_285380 |
|  | A/Jiangsu/11556/2017 | H7N9 | EPI_ISL_258000 |
|  | A/Hebei/27403/2017 | H7N9 | EPI_ISL_285161 |
|  | A/Environment/Hebei/27426/2017 | H7N9 | EPI_ISL_285039 |
|  | A/Beijing/28933/2017 | H7N9 | EPI_ISL_285012 |
|  | A/Environment/Hunan/03275/2015 | H9N2 | EPI_ISL_283819 |
|  | A/chicken/Zhejiang/SIC40/2015 | H9N2 | EPI_ISL_234464 |
|  | A/chicken/Guangdong/SZBAXQ008/2015(Mixed) | H9N2 | EPI_ISL_200605 |
|  | A/Hong Kong/4553/2016 | H7N9 | EPI_ISL_333971 |
|  | A/Guangdong/17SF060/2017 | H7N9 | EPI_ISL_285135 |
|  | A/Fujian/22/2014 | H7N9 | EPI_ISL_192322 |
|  | A/goose/Guangdong/A11/2016 | H9N2 | EPI_ISL_305607 |
|  | A/chicken/Guangdong/GD1601/2016 | H9N2 | EPI_ISL_284667 |
|  | A/environment/Zhongshan/ZS201505/2015 | H9N2 | EPI_ISL_235526 |
|  | A/chicken/Guangxi/SIC19/2014 | H9N2 | EPI_ISL_234434 |
|  | A/Chicken/Guangdong/GZ16491/2016 | H7N9 | EPI_ISL_249143 |
|  | A/chicken/Guangdong/SD1433/2016 | H7N9 | EPI_ISL_283501 |
|  | A/Environment/Guangdong/07103/2017 | H7N9 | EPI_ISL_285025 |
|  | A/guangdong/17SF017/2017 | H7N9 | EPI_ISL_285128 |
|  | A/Guangdong/CHN/023/2016 | H7N9 | EPI_ISL_239333 |
|  | A/Environment/Guangdong/16725/2017 | H9N2 | EPI_ISL_283959 |
|  | A/environment/Guangdong/C13280011/2013 | H9N2 | EPI_ISL_151431 |
|  | A/ostrich/Hebei/179/2014 | H9N2 | EPI_ISL_379147 |
|  | A/Yunnan/32294/2017 | H7N9 | EPI_ISL_285308 |
|  | A/environment/Zhongshan/ZS201503/2015 | H9N2 | EPI_ISL_235524 |
|  | A/environment/Zhongshan/ZS201603/2016 | H9N2 | EPI_ISL_235521 |
|  | A/Guangdong/18SF064/2018 | H9N2 | EPI_ISL_345234 |
|  | A/Guangdong/18SF003/2018 | H9N2 | EPI_ISL_337277 |
|  | A/Quail/Hong Kong/G1/97 | H9N2 | EPI_ISL_1268 |
|  | A/Duck/Hong Kong/702/79 | H9N2 | EPI_ISL_139 |
|  | A/Duck/Hong Kong/Y439/97 | H9N2 | EPI_ISL_1267 |
|  | A/Chicken/Hong Kong/G9/97 | H9N2 | EPI_ISL_1263 |
|  | A/Chicken/Beijing/1/94 | H9N2 | EPI_ISL_1270 |
| NCBI | A/chicken/Shanghai/1106-58/2017 | H9N2 | MG984011 |
|  | A/chicken/Shanghai/06/2015 | H9N2 | MK053855 |
|  | A/chicken/Zhejiang/HJ/2007 | H9N2 | FJ581432 |
|  | A/Hong Kong/1073/99 | H9N2 | AJ278647 |
|  | A/Guangzhou/333/99 | H9N2 | AY043025 |
|  | A/duck/Hong Kong/784/1979 | H9N2 | DQ107496 |
|  | A/Chicken/Korea/38349-p96323/96 | H9N2 | AF156467 |
|  | A/Turkey/California/189/66 | H9N2 | AF156471 |
|  | A/turkey/Wisconsin/1/1966 | H9N2 | DQ067438 |
|  | A/chicken/Heilongjiang/35/00 | H9N2 | DQ064393 |
|  | A/Chicken/Shanghai/F/98 | H9N2 | AY253755 |
|  | A/Pigeon/Hong Kong/Y233/97 | H9N2 | AF156460 |
|  | A/Duck/Hong Kong/Y280/97 | H9N2 | AF156461 |
|  | A/Swine/Hong Kong/9/98 | H9N2 | AF222822 |
|  | A/Chicken/Shandong/6/96 | H9N2 | DQ064403 |
|  | A/chicken/China/CK76/2017 | H9N2 | MN384729 |
|  | A/chicken/China/384/2017 | H9N2 | MN385391 |
|  | A/Chicken/Korea/25232-96006/96 | H9N2 | AF156468 |
|  | A/chicken/Xinjiang/01/2017 | H9N2 | MW100984 |
|  | A/chicken/Xinjiang/02/2017 | H9N2 | MW100985 |
|  | A/duck/Xinjiang/03/2017 | H9N2 | MW100986 |
|  | A/duck/Xinjiang/04/2017 | H9N2 | MW100987 |
|  | A/chicken/Xinjiang/05/2017 | H9N2 | MW106995 |
|  | A/chicken/Xinjiang/06/2017 | H9N2 | MW107003 |
|  | A/chicken/Xinjiang/07/2017 | H9N2 | MW100989 |
|  | A/chicken/Xinjiang/08/2017 | H9N2 | MW100990 |
|  | A/chicken/Xinjiang/09/2017 | H9N2 | MW100991 |
|  | A/chicken/Xinjiang/010/2017 | H9N2 | MW100992 |
|  | A/chicken/Xinjiang/011/2017 | H9N2 | MW106947 |
|  | A/chicken/Xinjiang/012/2017 | H9N2 | MW100993 |
|  | A/chicken/Xinjiang/013/2017 | H9N2 | MW100994 |
|  | A/chicken/Xinjiang/014/2017 | H9N2 | MW100995 |
|  | A/chicken/Xinjiang/015/2017 | H9N2 | MW106963 |
|  | A/chicken/Xinjiang/016/2017 | H9N2 | MW106955 |
|  | A/environment/Xinjiang/017/2017 | H9N2 | MW100996 |
|  | A/chicken/Xinjiang/018/2017 | H9N2 | MW100997 |
|  | A/chicken/Xinjiang/019/2017 | H9N2 | MW100998 |
|  | A/chicken/Xinjiang/020/2017 | H9N2 | MW100999 |
|  | A/chicken/Xinjiang/021/2017 | H9N2 | MW101000 |
|  | A/chicken/Xinjiang/022/2018 | H9N2 | MW101160 |
|  | A/chicken/Xinjiang/023/2018 | H9N2 | MW101161 |
|  | A/chicken/Xinjiang/024/2018 | H9N2 | MW106979 |
|  | A/chicken/Xinjiang/025/2018 | H9N2 | MW101162 |
|  | A/chicken/Xinjiang/026/2018 | H9N2 | MW101163 |
|  | A/chicken/Xinjiang/027/2018 | H9N2 | MW101164 |
|  | A/chicken/Xinjiang/028/2018 | H9N2 | MW101165 |
|  | A/duck/Xinjiang/029/2018 | H9N2 | MW101166 |
|  | A/chicken/Xinjiang/030/2018 | H9N2 | MW101167 |

| NS | Strain name | Subtype | Isolate_ID |
| --- | --- | --- | --- |
| GISAID | A/Xinjiang/04062/2018 | H7N9 | EPI_ISL_325939 |
|  | A/chicken/China/G861/2016 | H9N2 | EPI_ISL_501355 |
|  | A/Environment/Chongqing/38212/2016 | H9N2 | EPI_ISL_283723 |
|  | A/Environment/Shandong-Rizhao/34/2017 | H9N2 | EPI_ISL_283914 |
|  | A/Environment/Gansu/52450/2016 | H9N2 | EPI_ISL_283767 |
|  | A/Zhejiang/2/2017 | H7N9 | EPI_ISL_242855 |
|  | A/Environment/Hebei/13875/2017 | H9N2 | EPI_ISL_283813 |
|  | A/Environment/Xinjiang/32311/2017 | H7N9 | EPI_ISL_285092 |
|  | A/Environment/Inner-Mongolia/28665/2017 | H7N9 | EPI_ISL_285057 |
|  | A/Shanxi/26571/2017 | H7N9 | EPI_ISL_285271 |
|  | A/Environment/Jiangsu/12054/2016 | H9N2 | EPI_ISL_283863 |
|  | A/Hong-Kong/2550/2015 | H7N9 | EPI_ISL_170981 |
|  | A/Guangdong/15SF053/2015 | H7N9 | EPI_ISL_192284 |
|  | A/Environment/Guangdong/14925/2016 | H9N2 | EPI_ISL_283772 |
|  | A/Environment/Suzhou/sz21/2014 | H7N9 | EPI_ISL_170160 |
|  | A/guangdong/17SF017/2017 | H7N9 | EPI_ISL_285128 |
|  | A/chicken/China/G1773NS16GD/2016 | H9N2 | EPI_ISL_399136 |
|  | A/chicken/Guangdong/SZBJ0011-O/2017 | H7N9 | EPI_ISL_259758 |
|  | A/Hong-Kong/214/2017 | H7N9 | EPI_ISL_242275 |
|  | A/Shenzhen/Th002/2016 | H7N9 | EPI_ISL_250425 |
|  | A/Guangdong/18SF003/2018 | H9N2 | EPI_ISL_337277 |
|  | A/Guangdong/60061/2016 | H7N9 | EPI_ISL_242888 |
|  | A/chicken/China/G1333NS16GD/2016 | H9N2 | EPI_ISL_399127 |
|  | A/Guangdong/17SF032/2017 | H7N9 | EPI_ISL_267758 |
|  | A/Guangdong/Th008/2017 | H7N9 | EPI_ISL_250311 |
|  | A/Hunan/25362/2017 | H7N9 | EPI_ISL_285570 |
|  | A/Anhui/13439/2017 | H7N9 | EPI_ISL_258023 |
|  | A/guangdong/17SF011/2017 | H7N9 | EPI_ISL_285127 |
|  | A/chicken/China/G1737NS16GD/2016 | H9N2 | EPI_ISL_399134 |
|  | A/chicken/China/G1662NS16GD/2016 | H9N2 | EPI_ISL_399133 |
|  | A/chicken/China/a28/2017 | H9N2 | EPI_ISL_301879 |
|  | A/Henan/11158/2017 | H7N9 | EPI_ISL_258009 |
|  | A/chicken/Qingyuan/zd201601/2016 | H9N2 | EPI_ISL_378890 |
|  | A/chicken/Qingyuan/zd201602/2016 | H9N2 | EPI_ISL_378891 |
|  | A/Environment/Shandong-Laiwu/02/2017 | H9N2 | EPI_ISL_283908 |
|  | A/chicken/Shandong/1844/2019 | H9N2 | EPI_ISL_502323 |
|  | A/chicken/Shandong/1807/2019 | H9N2 | EPI_ISL_502320 |
|  | A/chicken/Shandong/FQ2/2019 | H9N2 | EPI_ISL_502326 |
|  | A/Beijing/25686/2017 | H9N2 | EPI_ISL_285004 |
|  | A/chicken/ShangDong/1624/2018 | H9N2 | EPI_ISL_368407 |
|  | A/chicken/Jiangsu/TM71/2014 | H9N2 | EPI_ISL_294490 |
|  | A/Beijing/1/2017 | H9N2 | EPI_ISL_285482 |
|  | A/chicken/Primorsky-Krai/03/2018 | H9N2 | EPI_ISL_321315 |
|  | A/chicken/Primorsky-Krai/1771/2018 | H9N2 | EPI_ISL_336927 |
|  | A/chicken/Anhui/LH99/2017 | H9N2 | EPI_ISL_327802 |
|  | A/chicken/ShangDong/16587/2018 | H9N2 | EPI_ISL_368402 |
|  | A/chicken/Zhejiang/HJ/2007 | H9N2 | EPI_ISL_63471 |
|  | A/Chicken/Shanghai/F/98 | H9N2 | EPI_ISL_68579 |
|  | A/chicken/Shandong/6/96 | H9N2 | EPI_ISL_2240 |
|  | A/Chicken/Hong-Kong/739/94 | H9N2 | EPI_ISL_1269 |
|  | A/Chicken/Beijing/1/94 | H9N2 | EPI_ISL_1270 |
|  | A/Duck/Hong-Kong/Y280/97 | H9N2 | EPI_ISL_1266 |
|  | A/Chicken/Hong-Kong/G9/97 | H9N2 | EPI_ISL_1263 |
|  | A/Chicken/Hong-Kong/G23/97 | H9N2 | EPI_ISL_1264 |
|  | A/Pigeon/Hong-Kong/Y233/97 | H9N2 | EPI_ISL_1265 |
|  | A/Quail/Hong-Kong/G1/97 | H9N2 | EPI_ISL_1268 |
|  | A/Hong-Kong/1073/99 | H9N2 | EPI_ISL_140 |
|  | A/Quail/Hong-Kong/AF157/92 | H9N2 | EPI_ISL_1271 |
|  | A/turkey/Wisconsin/1/1966 | H9N2 | EPI_ISL_70131 |
|  | A/Duck/Hong-Kong/Y439/97 | H9N2 | EPI_ISL_1267 |
|  | A/Chicken/Korea/38349-p96323/96 | H9N2 | EPI_ISL_1274 |
|  | A/Chicken/Korea/25232-96006/96 | H9N2 | AF156468 |
| NCBI | A/chicken/Anhui/AH450/2017 | H9N2 | MH489439 |
|  | A/chicken/Jiangsu/TM261/2017 | H7N9 | MG575621 |
|  | A/chicken/Japan/AQ-HE28-28/2016 | H9N2 | LC374927 |
|  | A/chicken/Yuhuan/YH15/2016 | H9N2 | KY415937 |
|  | A/duck/Wenzhou/YHQL64/2014 | H9N2 | KU143454 |
|  | A/chicken/China/384/2017 | H9N2 | MN385392 |
|  | A/chicken/China/355/2017 | H9N2 | MN385383 |
|  | A/Guangdong/SP440/2017 | H7N9 | KY643852 |
|  | A/chicken/Shandong/WF39/2016 | H9N2 | MN135937 |
|  | A/Changsha/58/2017 | H7N9 | MF370262 |
|  | A/chicken/Shanghai/1127-35/2017 | H9N2 | MG984032 |
|  | A/chicken/Jiangsu/TM315/2017 | H9N2 | MH489495 |
|  | A/chicken/Shandong/416/2016 | H9N2 | MN857557 |
|  | A/chicken/Xinjiang/01/2017 | H9N2 | MW101939 |
|  | A/chicken/Xinjiang/02/2017 | H9N2 | MW101940 |
|  | A/duck/Xinjiang/03/2017 | H9N2 | MW101941 |
|  | A/duck/Xinjiang/04/2017 | H9N2 | MW101942 |
|  | A/chicken/Xinjiang/05/2017 | H9N2 | MW106996 |
|  | A/chicken/Xinjiang/06/2017 | H9N2 | MW107004 |
|  | A/chicken/Xinjiang/07/2017 | H9N2 | MW101943 |
|  | A/chicken/Xinjiang/08/2017 | H9N2 | MW101944 |
|  | A/chicken/Xinjiang/09/2017 | H9N2 | MW101945 |
|  | A/chicken/Xinjiang/010/2017 | H9N2 | MW101946 |
|  | A/chicken/Xinjiang/011/2017 | H9N2 | MW106948 |
|  | A/chicken/Xinjiang/012/2017 | H9N2 | MW101947 |
|  | A/chicken/Xinjiang/013/2017 | H9N2 | MW101948 |
|  | A/chicken/Xinjiang/014/2017 | H9N2 | MW101949 |
|  | A/chicken/Xinjiang/015/2017 | H9N2 | MW106964 |
|  | A/chicken/Xinjiang/016/2017 | H9N2 | MW106956 |
|  | A/environment/Xinjiang/017/2017 | H9N2 | MW101950 |
|  | A/chicken/Xinjiang/018/2017 | H9N2 | MW101951 |
|  | A/chicken/Xinjiang/019/2017 | H9N2 | MW101952 |
|  | A/chicken/Xinjiang/020/2017 | H9N2 | MW101953 |
|  | A/chicken/Xinjiang/021/2017 | H9N2 | MW101954 |
|  | A/chicken/Xinjiang/022/2018 | H9N2 | MW102112 |
|  | A/chicken/Xinjiang/023/2018 | H9N2 | MW102113 |
|  | A/chicken/Xinjiang/024/2018 | H9N2 | MW106980 |
|  | A/chicken/Xinjiang/025/2018 | H9N2 | MW102114 |
|  | A/chicken/Xinjiang/026/2018 | H9N2 | MW102115 |
|  | A/chicken/Xinjiang/027/2018 | H9N2 | MW102116 |
|  | A/chicken/Xinjiang/028/2018 | H9N2 | MW102117 |
|  | A/duck/Xinjiang/029/2018 | H9N2 | MW102118 |
|  | A/chicken/Xinjiang/030/2018 | H9N2 | MW102119 |
