# Supplementary material for: Complex Reassortment Dynamics of H9N2 Avian Influenza Viruses in Xinjiang, China: Implications for Zoonotic Spillover
Source: Influenza Other Respir Viruses. 2025 Oct 20;19(10):e70170. doi: 10.1111/irv.70170 (PMC12537064; doi:10.1111/irv.70170)
Supplement: Supplementary file 2 — Table S2: Detailed information of the eight genes of the reference sequence used for phylogenetic analysis. [file IRV-19-e70170-s002.docx]

**Supplementary Table 2. Amino acid mutations identified in the H9N2 isolates contribute to increased viral binding to human-type receptors, replication, virulence, or transmission in mammals..**

| **Virus** | **The mutations that enhance the virus's affinity for human-type receptors in HA** | | | | |  | **Mutations that enhance viral replication, virulence, or transmission in mammalian hosts** | | | | | | | | | | | | | | | | | | | |  |
| --- | --- | --- | --- | --- | --- | --- | --- | --- | --- | --- | --- | --- | --- | --- | --- | --- | --- | --- | --- | --- | --- | --- | --- | --- | --- | --- | --- |
|  |  |  |  |  |  |  | **PB2** | | | | | |  | **PB1** | |  | | **PA** | | |  | **M1** | |  | **NS1** | | |
|  | I155T | H183N | T190A | Q226L | G228S |  | L89V | G309D | A588V/I | R477G | E627V | I495V |  | L13P | I368V | |  | K356R | N383D | A515T |  | N30D | T215A |  | P42S | V149A |  |
| XJ/01/17 | T | N | A | L | G |  | V | D | I | G | E | V |  | P | V | |  | R | D | T |  | D | A |  | S | A |  |
| XJ/02-03/17 | T | N | A | L | G |  | V | D | V | G | E | V |  | P | V | |  | R | D | T |  | D | A |  | S | A |  |
| XJ/04/17 | T | N | A | L | G |  | V | D | V | G | V | V |  | P | V | |  | R | D | T |  | D | A |  | S | A |  |
| XJ/05-017/17 | T | N | A | L | G |  | V | D | V | G | E | V |  | P | V | |  | R | D | T |  | D | A |  | S | A |  |
| XJ/018-021/17 | T | N | A | L | G |  | V | D | V | G | V | V |  | P | V | |  | R | D | T |  | D | A |  | S | A |  |
| XJ/022-024/18 | T | N | A | L | G |  | V | D | V | G | V | V |  | P | V | |  | R | D | T |  | D | A |  | S | A |  |
| XJ/025/18 | T | N | A | L | G |  | V | D | V | G | E | V |  | P | V | |  | R | D | T |  | D | A |  | S | A |  |
| XJ/026/18 | T | N | A | L | G |  | V | D | V | G | E | V |  | P | V | |  | R | D | T |  | D | A |  | S | A |  |
| XJ/027-30/18 | T | N | A | L | G |  | V | D | V | G | V | V |  | P | V | |  | R | D | T |  | D | A |  | S | A |  |
